# Dynamic regulatory on/off minimization for biological systems under internal temporal perturbations

**DOI:** 10.1186/1752-0509-6-16

**Published:** 2012-03-12

**Authors:** Sabrina Kleessen, Zoran Nikoloski

**Affiliations:** 1 Max-Planck Institute of Molecular Plant Physiology, 14476 Potsdam, Germany; 2 Institute of Biochemistry and Biology, University of Potsdam, 14476 Potsdam, Germany

## Abstract

**Background:**

Flux balance analysis (FBA) together with its extension, dynamic FBA, have proven instrumental for analyzing the robustness and dynamics of metabolic networks by employing only the stoichiometry of the included reactions coupled with adequately chosen objective function. In addition, under the assumption of minimization of metabolic adjustment, dynamic FBA has recently been employed to analyze the transition between metabolic states.

**Results:**

Here, we propose a suite of novel methods for analyzing the dynamics of (internally perturbed) metabolic networks and for quantifying their robustness with limited knowledge of kinetic parameters. Following the biochemically meaningful premise that metabolite concentrations exhibit smooth temporal changes, the proposed methods rely on minimizing the significant fluctuations of metabolic profiles to predict the time-resolved metabolic state, characterized by both fluxes and concentrations. By conducting a comparative analysis with a kinetic model of the Calvin-Benson cycle and a model of plant carbohydrate metabolism, we demonstrate that the principle of regulatory on/off minimization coupled with dynamic FBA can accurately predict the changes in metabolic states.

**Conclusions:**

Our methods outperform the existing dynamic FBA-based modeling alternatives, and could help in revealing the mechanisms for maintaining robustness of dynamic processes in metabolic networks over time.

## Background

Systems biology paradigm has provided insights in the maintenance of robustness for biological processes involving a multitude of interconnected elements (e.g., genes, proteins, metabolites) [1]. In addition, recent advances in metabolomics have provided a large amount of highly reproducible data [2-4], allowing reconstruction and analysis of genome-scale metabolic networks [5]. These developments in metabolomics technologies have challenged computational systems biology with the need to accurately describe the dynamics of metabolic networks in order not only to glean the flux rates at different time points, representing the temporal flux (re)distribution, and the interdependent metabolic profiles, but also to identify key elements for metabolic engineering [6-10].

Metabolic flux analysis (MFA) has propelled the development of computational methods for analysis of metabolic networks [11,12]. Flux balance analysis (FBA), as one of the most prominent of the MFA methods, is based on linear programming (LP) whereby a given objective function (e.g., biomass yield) is optimized under the assumption that the system operates at steady state under the constraints given by the stoichiometric matrix [11,13-16]. By optimizing an objective function, the linear program identifies one feasible flux distribution from the set of fluxes satisfying the constraints imposed by the mass-balance equations and reaction bounds [15]. Consequently, the biological implications of the optimal flux distribution depend on the choice of the objective function [17]. Maximization of biomass is one of these functions, which is assumed particularly suitable for microbial models [18]. For eukaryotic cells (e.g., in plants) where biomass or yield may not be the primary goal, a different objective function has to be determined. For instance, cellular maintenance at minimal efforts has been proposed to be one of the alternatives [19]. Nevertheless, finding an adequate objective of a metabolic network, and especially a sub network related to particular metabolic processes, remains a problem of ongoing interest [18,20]. However, the steady-state assumption on which FBA is based precludes the analysis of the dynamics of metabolite concentrations and flux (re)distribution. Furthermore, the classical FBA ignores the possibility that perturbed metabolic networks may not immediately regulate towards the (assumed) optimal objective.

Based on the hypothesis that fluxes in metabolic networks, altered by removal of a reaction, undergo a minimal redistribution compared to those of the wild type, minimization of metabolic adjustment (MOMA) [21] and regulatory on/off minimization (ROOM) [22] have been devised as two contending alternatives for analysis of perturbed metabolic network models. MOMA predicts the flux redistribution which has the smallest Euclidean distance to the wild type flux distribution obtained by FBA, while ROOM minimizes the number of (significant) flux changes from the wild type flux distribution. As a consequence, large modifications in single fluxes are prevented in MOMA; however, such large modifications may be required for rerouting metabolic flux through alternative pathways [22], which has been observed in experiments [23]. Existing studies have demonstrated that ROOM outperforms MOMA and FBA in the flux prediction of the final metabolic steady state, albeit, in the particular case of pyruvate kinase knockout in *E. coli *[22].

The analysis of metabolic network dynamics has traditionally been facilitated by models based on ordinary differential equations (ODEs) which require a large amount of information for simulating the temporal metabolic changes [9,10]. To this end, the phenomenological parameters of specific enzyme kinetics (e.g., Michaelis-Menten or Hill) have to be determined by accurate measurements of enzyme activities and data-fitting to experimentally obtained (time-course) data. In turn, the obtained fits are often used to make predictions and draw conclusions based on the postulated kinetic model.

On the other hand, dynamic FBA (DFBA) offers the alternative to predict time-resolved metabolic profiles with limited knowledge of enzyme kinetics [6-8]. Unlike, the analyses based on FBA, which focus on the steady-state behavior, DFBA offers the means to analyze transient (non-steady) states. In addition, DFBA has been combined with MOMA, resulting in the M-DFBA approach which has been employed for predicting the dynamics of photosynthetic metabolism and positing hypotheses about its robustness under different CO_2_ and water conditions [7]. M-DFBA extends the MOMA hypothesis from minimal redistribution of metabolic fluxes in perturbed metabolic networks to a minimal fluctuation of the profile of metabolite concentrations over time.

We point out that the mechanisms describing the *temporal *changes in the metabolic state, characterized by the metabolite concentrations and flux rates, are principal to the notion of robustness, as used with DFBA-based approaches. For instance, the posited mechanism in M-DFBA is that metabolic networks operate to minimize the fluctuations in metabolic concentrations over time. This suggests alterations to the definition of robustness as a property that maintains system function in the case of *external *perturbations [24,25] to include the *internal *perturbations due to changes in the dynamic state of the system's elements. External perturbations arise due to changing environmental conditions, such as stress conditions (environmental perturbations), but also by changes in the structure of a metabolic networks (e.g., caused by gene knock-outs, also known as genetic perturbations). In contrast, internal perturbations are due to temporal changes of the metabolic state characterized by the coupling of metabolite concentrations and flux distributions. Considering mass actions kinetics, which states that the flux rate of a reaction is proportional to the product of the concentrations of the participating reactants [26]. As a result, even in this simplest kinetic law, a change in metabolite concentrations may affect flux rates, which, in turn, as a result of the mass balances have an effect on the concentrations. Here, we investigate a set of biochemically plausible hypotheses which can be used to characterize the mechanisms responsible for maintaining the metabolic network robustness due to internal perturbations. These mechanisms can in turn be used to simulate the dynamics of a given metabolic network relying purely on the stoichiometry and a limited amount of information regarding the phenomenological constants.

Driven by the idea of mutually influencing system elements, central to the systems biology paradigm, we argue that minimal fluctuations of metabolic profiles may only represent one possible explanation of the temporal changes in metabolic state. In this study, we design the ROOM-based DFBA approach (abbreviated to R-DFBA) by coupling the principle of ROOM with DFBA. R-DFBA extends the premise of the ROOM approach, which relies on significant flux changes, by considering the minimization of the total number of significant changes of metabolite concentrations. Furthermore, the coupling of ROOM and DFBA renders it possible to use the advantages of ROOM compared to MOMA in a dynamic setting.

In addition, we modify and extend the proposed R-DFBA and the existing M-DFBA approaches to consider minimizing fluctuations not only in concentrations but also in flux levels, which result in **seven **proposed methods based on different optimization functions and programming formulations. Including classical DFBA and the two known variants of M-DFBA [8], a total of ten different approaches are presented to predict and analyze the dynamics of metabolite concentrations and flux levels over time. Finally, the accuracy of a dynamic FBA-based approach in simulating the dynamics of metabolic networks must be established based on the comparison of the obtained results and the outcome of a well-defined kinetic model for a given metabolic network. Here, by comparing the results of applying DFBA, M-DFBA, R-DFBA and their extensions to those of the kinetic models for the Calvin-Benson cycle and plant carbohydrate metabolism, our analysis discriminates between the different mechanisms which result in the apparent metabolic network dynamics and robustness. Our quantitative and qualitative comparative analyses demonstrate that the extensions based on R-DFBA outperform the existing DFBA-based approaches.

## Results and discussion

**Figure 1:**
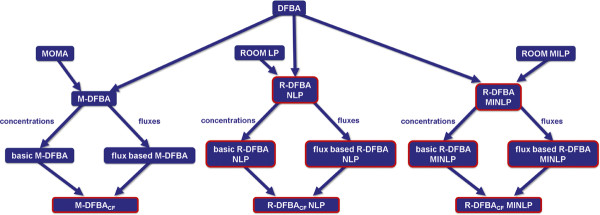
**Overview of the ten approaches, which are used to analyze the dynamics of metabolite and flux profiles in the Calvin-Benson cycle and the plant carbohydrate metabolism.** The novel methods proposed in this study are framed in red.

Here we describe the seven proposed methods and the comparison of their performance with two kinetic models--of the Calvin-Benson cycle and of the plant carbohydrate metabolism. Since the proposed methods build upon the DFBA approaches, we provide a brief overview of the mathematical apparatus used in their formulation. The suite of proposed methods and their relation to the existing DFBA approaches are depicted in Figure 1.

We point out that DFBA-based methods and kinetic modeling constitute independent approaches. The DFBA-based methods involve performing a constrained optimization over a time period to approximate the dynamics of a system with stoichiometric constraints only, which include relative quantities of reactants and products of the particular reaction. In contrast, a kinetic model requires all kinetic parameters to simulate the dynamics of the system. However, kinetic modeling is often hampered by incomplete knowledge of the underlying enzyme-kinetics and their associated parameter values [17].

In addition, it is necessary to determine the extent to which the dynamics of both metabolite profiles as well as flux predictions can be successfully predicted from DFBA-based approaches. The outcome of testing this hypothesis will then suggest the most likely mechanism which describes the robustness of the system to internal perturbations over time. We argue that such approach goes beyond the analysis of steady states typical for metabolic control analysis (MCA) [27] and structural kinetic modeling as its extension [28].

### DFBA

DFBA overcomes the main drawback of the classical FBA which precludes the analysis of the dynamic behavior of a network-the steady-state assumption. Mahadevan and coworkers introduced two DFBA formulations--static and dynamic [6]. The static optimization (SOA) involves first dividing the time period of interest into uniform time intervals and then solving the instantaneous optimization problem at the beginning of each time interval, followed by integration to compute the metabolite concentration over time. In contrast, the general dynamic optimization approach (DOA) is formulated as follows:

$$\begin{array}{c} \text{max}\overset{t_{f}}{\underset{t_{0}}{\int }}f\left(x\right)dx\\ \text{s.t.}\frac{dX}{dt}=S\cdot v\\ v_{min}\leq v\leq v_{max}\\ x_{min}\leq x\leq x_{max}\\ X\left(t_{0}\right)=X_{0}, \end{array}$$

where *x *and *v *are vectors of metabolite concentrations and reaction fluxes over time, *S *denotes the stoichiometric matrix, with rows corresponding to metabolites and columns to reactions of the metabolic network described by *S*, and *t *is the time. The minimum and maximum allowable fluxes of each reaction and metabolite concentrations are defined by *v*_*min *_and *v*_*max *_and *x*_*min *_and *x*_*max*_, respectively. The vector *X*_0 _gives the initial concentration for the set of metabolites. The formulation of the DOA approach results in a nonlinear program (NLP) if nonlinear constraints and/or a nonlinear objective function are included.

The DOA involves optimization over the entire time period *t *to obtain time-resolved flux rates and metabolite concentrations. The optimization is rendered computationally feasible by parameterizing the dynamic equations with the help of orthogonal collocation on finite elements [29]. To this end the time period of interest is divided into a finite number of intervals, named *finite elements*. Furthermore, the metabolite concentrations *x *and flux levels v are parameterized at the roots of an orthogonal polynomial (e.g., Legendre polynomial) within each finite element [6]. For readers not familiar with orthogonal collocation on finite elements an instructive example of how this algorithm works is provided in Additional file 1.

We point out that the number of variables in each optimization step of SOA is smaller compared to that of DOA, allowing scalability to larger networks. However, SOA does not allow dynamic formulation, and the remainder of the methods focus on DOA.

### M-DFBA

Luo and coworkers developed an approach called M-DFBA which combines MOMA with DFBA [7]. By employing the MOMA hypothesis, the objective function of the dynamic optimization approach of DFBA is altered to a minimization of the Euclidean distance between metabolite concentrations at adjacent orthogonal roots $\sqrt{{\sum_{i=1}^{N}\left(x_{i,j}-x_{i,j-1}\right)}^{2}}$, where *N *is the number of metabolites in the network and *x*_*i,j *_represents the concentration of metabolite *i *at the time point given by the orthogonal root *j*. Hence, the general M-DFBA optimization problem is defined as follows:

$$\begin{array}{c} \underset{x}{\text{min}}\overset{M}{\underset{j=1}{\sum }}\overset{t_{f}}{\underset{t_{0}}{\int }}\left(\sqrt{\overset{N}{\underset{i=1}{\sum }}{\left(x_{i,j}-x_{i,j-1}\right)}^{2}}\right)\delta \left(t-t_{j}\right)dt\\ \text{s.t.}\frac{dX}{dt}=S\cdot v\\ v_{min}\leq v\leq v_{max}\\ x_{min}\leq x\leq x_{max}\\ X\left(t_{0}\right)=X_{0} \end{array}$$

with *M *representing the number of orthogonal roots, and δ, the Dirac delta function (see Additional file 1).

### Extended versions of M-DFBA

We extend the M-DFBA method so that the objective function, subject to minimization, is given by the sum of Euclidean distances between metabolite concentrations and reaction rates at adjacent orthogonal roots:

$$\underset{x,v}{\text{min}}\overset{M}{\underset{j=1}{\sum }}\overset{t_{f}}{\underset{t_{0}}{\int }}\left(\sqrt{\overset{N}{\underset{i=1}{\sum }}{\left(x_{i,j}-x_{i,j-1}\right)}^{2}}+\sqrt{\overset{F}{\underset{l=1}{\sum }}{\left(v_{l,j}-v_{l,j-1}\right)}^{2}}\right)\delta \left(t-t_{j}\right)dt,$$

where *F *represents the number of reactions in the network. This approach, denoted M-DFBA*_CF_*, extends the M-DFBA approach by considering the hypothesis of the minimal fluctuation of flux levels in addition to minimal fluctuation of the profile of metabolite concentrations. In general, only limited knowledge of the enzyme-kinetic rate laws is available for an incorporation of kinetic expressions in the constraints. On the other hand, the inclusion of only stoichiometric constraints for the majority of the reactions can have the effect that flux rates and metabolite concentrations do not change as expected due to their coupling based on the underlying kinetics. Therefore, stabilizing only the profile of metabolite concentrations in the objective function, like in the basic M-DFBA approach, may lead to a large variation of flux rates over time. The inclusion of both metabolite concentrations and flux rates in the objective function renders a more constrained optimization problem, which could avoid large fluctuations in the predictions.

Moreover, to facilitate discrimination of the different mechanisms yielding a particular time-resolved metabolic state, we consider a third type of the M-DFBA method, whereby the objective function, subject to minimization, includes only the fluctuation of flux rates, as follows:

$$\underset{v}{\text{min}}\overset{M}{\underset{j=1}{\sum }}\overset{t_{f}}{\underset{t_{0}}{\int }}\left(\sqrt{\overset{F}{\underset{l=1}{\sum }}{\left(v_{l,j}-v_{l,j-1}\right)}^{2}}\right)\delta \left(t-t_{j}\right)dt.$$

### R-DFBA

In contrast to MOMA, ROOM minimizes the total number of significant (large enough) flux changes from the wild-type flux distribution. In our approach, we combine ROOM with DFBA, naming this modeling approach R-DFBA. Here, the ROOM hypothesis is extended from a minimization of significant flux changes to a minimization of fluctuation of the profile of metabolite concentrations, according to the assumption of the basic M-DFBA approach. Unlike M-DFBA, in R-DFBA this is realized by minimizing the significant concentration changes at adjacent orthogonal roots. In contrast to M-DFBA, which captures smooth changes over time (by the Euclidean distance), R-DFBA prevents a large number of small (significant) changes over time. Instead, it allows a large concentration change at a few time points (orthogonal roots) and a relatively constant concentration of the metabolites elsewhere, which is bounded by thresholds determining the significant change. Finally, there are two possibilities to capture concentration changes between two time points--the continuous, specified by the usage of a distance measure (e.g., Euclidean distance), and the binary, based on an appropriate definition of significant change. The proposed formulation of R-DFBA captures the binary case, as the remaining alternative to specify dynamic changes. From a biological perspective, the possibility for large concentration changes in a small time range, as specified in R-DFBA, may be necessary to capture the difference in concentrations between two steady states in a bi-stable system [30-32].

Two different types of the R-DFBA approach are considered: The first includes the basic ROOM approach with integer constraints, whereas the second relies on relaxing the integer constraints. Due to the relaxation of the integer constraints, the programming problem in ROOM becomes linear in comparison to mixed-integer linear program (MILP) in the basic ROOM approach [22]. The first R-DFBA approach, based on MILP ROOM, is described as follows:

$$\begin{array}{c} \underset{y}{\text{min}}\overset{M}{\underset{j=1}{\sum }}\overset{t_{f}}{\underset{t_{0}}{\int }}\overset{N}{\underset{i=1}{\sum }}y_{i,j}\delta \left(t-t_{j}\right)dt\\ \text{s.t.}\frac{dX}{dt}=S\cdot v\\ v_{min}\leq v\leq v_{max}\\ x_{min}\leq x\leq x_{max}\\ X\left(t_{0}\right)=X_{0}\\ \forall j,i1\leq j\leq M,1\leq i\leq N\\ x_{i,j}-y_{i,j}\left(x_{max,i}-w_{i,j}^{u}\right)\leq w_{i,j}^{u}\\ x_{i,j}-y_{i,j}\left(x_{min,i}-w_{i,j}^{l}\right)\geq w_{i,j}^{l}\\ y_{i,j}\in \left\{0,1\right\}\\ w_{i,j}^{u}=x_{i,j-1}+\gamma_{x}\left|x_{i,j-1}\right|+\varepsilon_{x}\\ w_{i,j}^{l}=x_{i,j-1}-\gamma_{x}\left|x_{i,j-1}\right|-\varepsilon_{x}, \end{array}$$

where *w*^*u*^, *w*^*l *^are the thresholds determining significant changes of metabolite concentrations (*u *= upper bound, *l *= lower bound) and *γ*_*x*_, *ε*_*x *_are the relative and absolute ranges of tolerance, respectively.

Due to the constraints defining the thresholds, the programming problem becomes a mixed-integer nonlinear programming problem (MINLP). These problems are difficult to solve precisely as they couple the combinatorial nature of mixed-integer programming (MIP) problem and the computational complexity of solving NLP problems [33].

Consequently, we formulate a second R-DFBA approach based on relaxed integer constraints, described as follows:

$$\begin{array}{c} \underset{y}{\text{min}}\overset{M}{\underset{j=1}{\sum }}\overset{t_{f}}{\underset{t_{0}}{\int }}\overset{N}{\underset{i=1}{\sum }}y_{i,j}\delta \left(t-t_{j}\right)dt\\ \text{s.t.}\frac{dX}{dt}=S\cdot v\\ v_{min}\leq v\leq v_{max}\\ x_{min}\leq x\leq x_{max}\\ X\left(t_{0}\right)=X_{0}\\ \forall j,i1\leq j\leq M,1\leq i\leq N\\ x_{i,j}-y_{i,j}\left(x_{max,i}-x_{i,j-1}\right)\leq x_{i,j-1}\\ x_{i,j}-y_{i,j}\left(x_{min,i}-x_{i,j-1}\right)\geq x_{i,j-1}\\ 0\leq y_{i,j}\leq 1. \end{array}$$

Compared to the first formulation of R-DFBA, the relative and absolute ranges of tolerance (*γ*_*x*_, and *ε*_*x*_) are set to be zero, due to the relaxed integer constraints for *y*_*i,j*_.

### Extended versions of R-DFBA

As for the M-DFBA approach, we also extend the proposed R-DFBA by considering the minimization of fluctuation of flux levels in addition to the minimized fluctuation of the profile of metabolite concentrations at adjacent orthogonal roots. We denote this approach as R-DFBA_*CF*_:

$$\begin{array}{c} \underset{y,z}{\text{min}}\overset{M}{\underset{j=1}{\sum }}\overset{t_{f}}{\underset{t_{0}}{\int }}\left(\overset{N}{\underset{i=1}{\sum }}y_{i,j}+\overset{F}{\underset{l=1}{\sum }}z_{l,j}\right)\delta \left(t-t_{j}\right)dt\\ \text{s.t.}\frac{dX}{dt}=S\cdot v\\ v_{min}\leq v\leq v_{max}\\ x_{min}\leq x\leq x_{max}\\ X\left(t_{0}\right)=X_{0}\\ \forall j,i,l\;1\leq j\leq M,1\leq i\leq N,1\leq l\leq F\\ x_{i,j}-y_{i,j}\left(x_{max,i}-w_{i,j}^{u}\right)\leq w_{i,j}^{u}\\ x_{i,j}-y_{i,j}\left(x_{min,i}-w_{i,j}^{l}\right)\geq w_{i,j}^{l}\\ y_{i,j}\in \left\{0,1\right\}\\ w_{i,j}^{u}=x_{i,j-1}-\gamma_{x}\left|x_{i,j}\right|-\varepsilon_{x}\\ w_{i,j}^{l}=x_{i,j-1}-\gamma_{x}\left|x_{i,j}\right|-\varepsilon_{x}\\ v_{l,j}=z_{l,j}\left(v_{max,l}-b_{l,j}^{u}\right)\leq b_{l,j}^{u}\\ v_{l,j}=z_{l,j}\left(v_{min,l}-b_{l,j}^{l}\right)\geq b_{l,j}^{l}\\ z_{l,j}\in \left\{0,1\right\}\\ b_{l,j}^{u}=v_{l,j-1}+\gamma_{v}\left|v_{l,j-1}\right|+\varepsilon_{v}\\ b_{l,j}^{l}=v_{l,j-1}-\gamma_{v}\left|v_{l,j-1}\right|-\varepsilon_{v}, \end{array}$$

where *b*^*u*^, *b*^*l *^are the thresholds determining significance of the flux levels (*u *= upper bound, *l *= lower bound) and *γ*_*v*_, *ε*_*v *_are the relative and absolute ranges of tolerance, respectively. Due to the inclusion of the flux rates and metabolite concentrations in the description of the thresholds, R-DFBA_*CF *_is solved based on mixed-integer nonlinear programming, by the first R-DFBA formulation, and based on nonlinear programming, according to the second. This extension minimizes fluctuations of both, metabolite concentrations and flux rates, over time, similarly to M-DFBA_*CF*_. In comparison, in R-DFBA_*CF *_large concentration and flux rate changes between two adjacent orthogonal roots for some metabolites and/or reactions are possible, which is precluded in M-DFBA_*CF*_.

Additionally, we consider a third objective function for the R-DFBA approach, whereby only fluctuation of the profile of flux levels of the network over time is minimized:

$$\begin{array}{c} \underset{z}{\text{min}}\overset{M}{\underset{j=1}{\sum }}\overset{t_{f}}{\underset{t_{0}}{\int }}\left(\overset{F}{\underset{l=1}{\sum }}z_{l,j}\right)\delta \left(t-t_{j}\right)dt\\ \text{s.t.}\frac{dX}{dt}=S\cdot v\\ v_{min}\leq v\leq v_{max}\\ x_{min}\leq x\leq x_{max}\\ X\left(t_{0}\right)=X_{0}\\ \forall j,l1\leq j\leq M,1\leq l\leq F\\ v_{l,j}-z_{l,j}\left(v_{max,l}-b_{l,j}^{u}\right)\leq b_{l,j}^{u}\\ v_{l,j}-z_{l,j}\left(v_{min,l}-b_{l,j}^{l}\right)\geq b_{l,j}^{l}\\ z_{l,j}\in \left\{0,1\right\}\\ b_{l,j}^{u}=v_{l,j-1}+\gamma_{v}\left|v_{l,j-1}\right|+\varepsilon_{v}\\ b_{l,j}^{l}=v_{l,j-1}-\gamma_{v}\left|v_{l,j-1}\right|-\varepsilon_{v}, \end{array}$$

The proposed optimization functions of the MINLP and NLP variants of R-DFBA, flux-based R-DFBA and R-DFBA_*CF *_allow a comparison of the necessity of stabilizing the system's profile of concentration, flux or a combination of both to predict dynamics in a metabolic network.

### Comparison of the proposed methods and a kinetic model of the Calvin-Benson cycle

**Figure 2:**
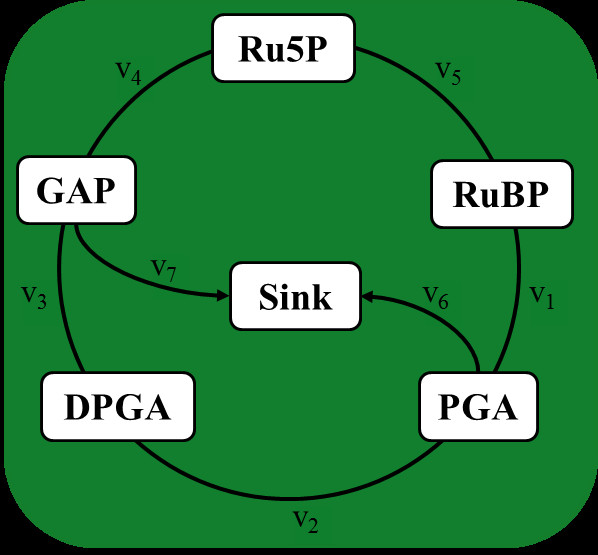
**Simplified model of the Calvin cycle**. The model includes six metabolites (Ru5P, RuBP, PGA, DPGA, GAP, and Sink) and seven reactions (v_1 _- v_7_).

The Calvin-Benson cycle consists of three phases relying on energy supply in form of *ATP *and redox elements *(NADP/NADPH) *and supply of CO_2_: (1) *carboxylation*, during which the enzyme RuBisCo adds CO^2 ^to ribulose-1,5-bisphosphate *(RuBP) *to get two molecules of phosphoglycerate *(PGA)*, (2) *reduction*, converting the obtained *PGA *into 1,3-diphosphoglycerate *(DPGA) *and glyceraldehyde-3-phosphate *(GAP)*, and (3) *regeneration*, which recovers *RuBP *after several intermediate steps from ribulose-5-phosphate *(Ru5P) *[34,35]. The Calvin-Benson cycle is a well-studied system of the plant metabolism due to its importance in providing precursors for the biomass synthesis which is necessary for plant growth. More than 15 kinetic models were already developed to analyze the dynamics of this important pathway [36]. In this study, we use the simplified model of Zhu et al. [37] to describe the dynamic behavior of the Calvin cycle, depicted in Figure 2 and Table 1. The concentrations of ATP, NADPH, phosphate (P), and CO_2 _are assumed to be positive and unbounded, yielding the simplified equations shown in Table 1[38]. For the present analysis, other factors which affect photosynthetic metabolism, such as illumination and temperature, are assumed to be constant.

**Table 1:** Reactions in the simplified model of the Calvin cycle

Symbol Enzyme name		Biochemical reaction	Simplified reaction	v_*max*_
v_1_	RuBisCo	RuBP + CO_2_ → 2 PGA	RuBP → 2 PGA	3.78
v_2_	PGA kinase	PGA + ATP → DPGA + ADP	PGA → DPGA	11.75
v_3_	GAP dehydrogenase	DPGA + NADPH → GAP + P + NADP	DPGA → GAP	5.04
v_4_		GAP → 0.6 Ru5P	GAP → 0.6 Ru5P	3.05
v_5_	Ru5P kinase	Ru5P + ATP → RuBP + ADP	Ru5P → RuBP	8
v_6_	Sink capacity	PGA → Sink	PGA → Sink	3
v_7_	Sink capacity	GAP → Sink	GAP → Sink	0.1

With the simplified model of the Calvin cycle, we examine and compare the performance of the proposed R-DFBA approach together with its extensions, the existing M-DFBA approach and our proposed extensions, as well as the classical DFBA. In total, ten different approaches, based on different constraint-based formulations, are used in the analysis of the dynamics of metabolite and flux profiles in the Calvin cycle (Figure 1). With each approach, the concentration of the metabolites ribulose-5-phosphate (Ru5P), ribulose-1,5-bisphosphate (RuBP), 3-phosphoglycerate (PGA), 1,3-diphosphoglycerate (DPGA), glyceraldehyde-3-phosphate (GAP) and sink as well as the rate of the seven reactions (v_1_-v_7_) are investigated over a time period of ten seconds, which is sufficient due to the fast settling of the system in a steady state [39].

For the analysis, the time period is divided into five finite elements, and the variables (metabolite concentrations and reaction rates) are parameterized at the roots of the fifth order Legendre polynomial. Maximizing the sink production is assumed as systemic objective for the DFBA approach, capturing the utilization of PGA and GAP to build the metabolite precursors necessary for sucrose and starch synthesis. For the remaining methods, the objective is modified to minimizing the fluctuation of the profile of metabolite concentrations/fluxes, according to the hypotheses of M-DFBA and R-DFBA.

The results obtained by the different methods are compared to the outcome of a kinetic model of the Calvin cycle, described in [37]. For the kinetic model as well as for the other compared methods, the same initial conditions are used (RuBP = 2.0 mmol l^-1^; PGA = 2.4 mmol l^-1^; DPGA, GAP, Ru5P, sink = 1.0 mmol l^-1^). In addition, for the DFBA-based approaches the limits for the GAP concentration over time are set to the outcome of the kinetic model with an added tolerance of +/- 0.1 mmol l^-1^. The solutions to MINLP formulation of R-DFBA (and its extensions) are obtained with *γ*_*x *_= 0.4, *γ*_*v *_= 0.2, and *ε*_*x *_= 0.01, *ε*_*v *_= 0.05, representing the relative and absolute ranges of tolerance, respectively. For each approach, we determine the residual sum of squares (RSS), quantifying the correspondence between the constraint-based and kinetic modeling approaches. Furthermore, we determine the temporal evolution of Kendall τ for the metabolic states of the constraint-based and kinetic modeling approaches in order to qualitatively examine the coupling between metabolic profiles and distribution of fluxes for the two types of modeling approaches.

#### R-DFBA yields accurate time-resolved predictions of metabolite profiles

**Figure 3:**
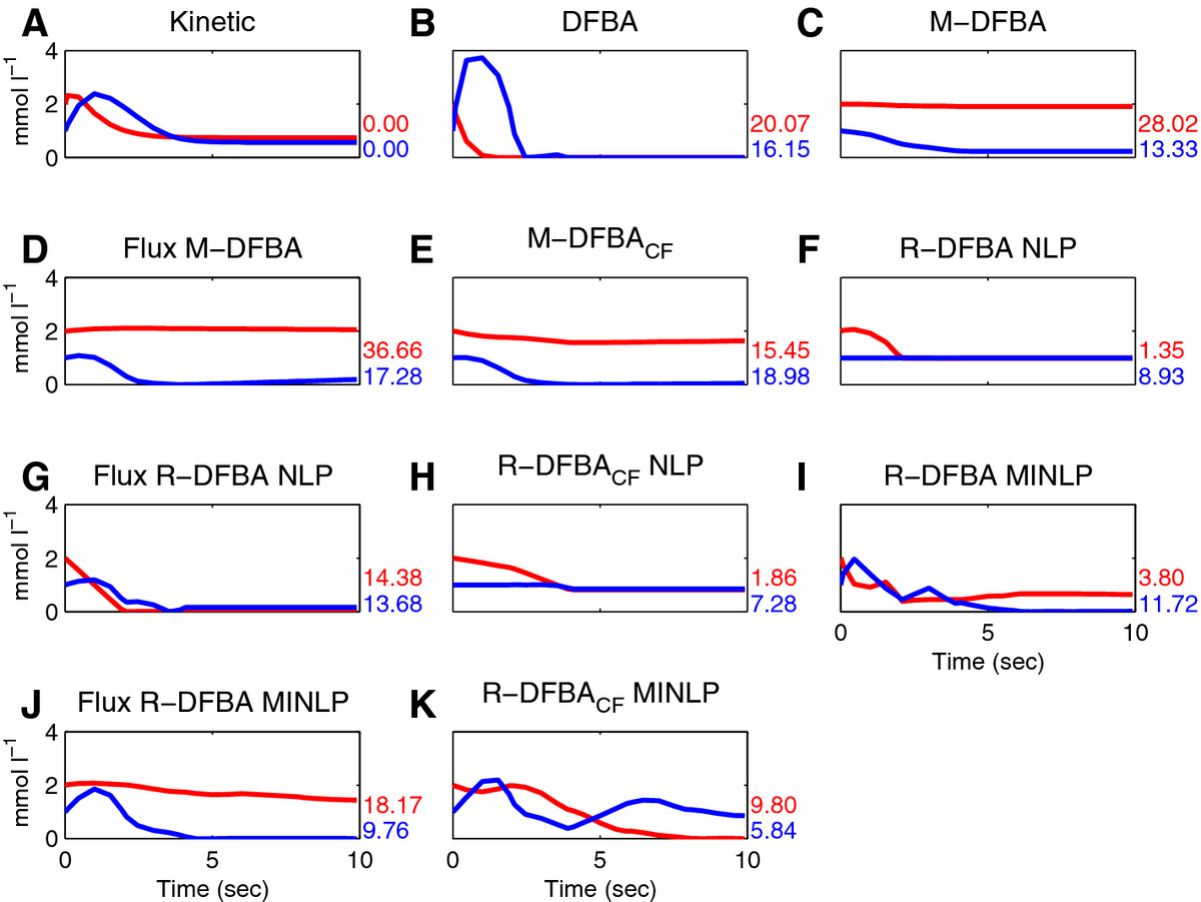
**Modeling results of concentrations of RuBP (red) and DPGA (blue) in the simplified model of the Calvin cycle by the different approaches**. The residual sum of squares values of each approach and metabolite are presented in the bottom right of the corresponding subfigure.

Figure 3 shows the simulations results of two metabolites, RuBP and DPGA, for the ten compared methods. For the proposed basic R-DFBA approach, minimizing the concentration fluctuations, the results differ between the variants: while the NLP-based variant predicts that the concentration of DPGA are almost constant over time, the MINLP-based formulation results in changes of the concentration of DPGA over the 10-second time interval. The results of basic M-DFBA show that the concentration of DPGA slightly decreases during the first three seconds, and is almost constant following this time period.

The effect of minimizing the flux changes is especially pronounced in the two variants of the flux-based R-DFBA. Here, the results for the DPGA concentration are similar to those of the basic M-DFBA and its extensions. The behavior of the DPGA concentration predicted by both flux-based R-DFBA variants at the beginning of the simulation is very close to the kinetic modeling results, which can be explained by the coupling between fluxes and concentrations giving rise to particular temporal profiles.

However, this behavior is lost in (NLP-based) R-DFBA_*CF*_, which combines the basic and flux-based variant of R-DFBA. In the variants of R-DFBA_*CF*_, the changes in flux rates and metabolite concentrations are weighted equally, as given by the objective function: $\sum_{i=1}^{N}y_{i,j}+\sum_{l=1}^{F}z_{l,j}$. A different weighting in the objective function of R-DFBA_*CF *_could avoid the loss of the observed behavior. This suggest that the objective function may have a more intricate form (e.g., $\sum_{i=1}^{N}\alpha y_{i,j}+\sum_{l=1}^{F}\left(1-\alpha \right)z_{l,j}$, where α is a parameter weighting the contribution of reaction rates and metabolite concentrations).

The results from the DFBA approach for the DPGA and RuBP concentration over time are very similar, with small changes in the first three seconds, leading to almost zero concentrations at the end of the simulation. The predicted RuBP concentrations of the basic M-DFBA and its extensions are nearly constant over time; on the other hand, with the proposed NLP-based R-DFBA approach, changes in concentrations are obtained in the first half of the simulated time interval, followed by a constant concentration. The concentration of this metabolite at the end of the simulation differs between the proposed MINLP-based R-DFBA variants. We point out that experimental measurements in isolated cells and chloroplasts have resulted in low levels of RuBP [39-41].

Comparing these results with the outcome of kinetic modeling shows that for these two metabolites MINLP-based R-DFBA predicts the most accurate temporal profiles. The constant concentration in the second half of the simulated interval of the NLP-based R-DFBA is very close to that of kinetic modeling, for both, the basic R-DFBA and R-DFBA_*CF*_. To quantify these observations, we determine the residual sum of squares, by calculating the distance between kinetic modeling results and predicted results of the different approaches, see Table 2. The proposed approaches exhibit the smallest RSS values for these two metabolites, demonstrating the predictive power of R-DFBA not only on MINLP but also on NLP.

**Table 2:** Residual sum of squares values for each metabolite

Method	Metabolite					
	RuBP	PGA	DPGA	GAP	Ru5P	Sink
DFBA	20.07	2.21	16.15	2.55	3.55	224.86
M-DFBA	28.02	7.58	13.33	2.54	3.20	567.42
Flux-based M-DFBA	36.66	2.32	17.28	2.22	3.57	534.20
M-DFBA_*CF*_	15.45	28.17	18.98	2.15	3.54	564.26
R-DFBA NLP	1.35	76.20	8.93	3.91	22.24	582.23
Flux-based R-DFBA NLP	14.38	345.25	13.68	2.35	5.66	558.03
R-DFBA_*CF *_NLP	1.86	14.97	7.28	3.57	23.53	582.23
R-DFBA MINLP	3.80	67.25	11.72	3.17	0.61	129.49
Flux-based R-DFBA MINLP	18.17	0.66	9.76	2.50	18.85	497.71
R-DFBA_*CF *_MINLP	9.80	5.17	5.84	3.01	55.48	420.66

The discrepancy between the kinetic modeling results and the results obtained from the DFBA-based approaches is quantified based on the residual sum of squares

The simulated results of the concentrations of the other four metabolites are depicted in Figures S1 - S4 in Additional file 2. Due to the fact that we have integrated rather strict constraints for GAP, the predicted concentrations of this metabolite over time are very similar for all approaches and also compared to the kinetic-based model. For Ru5P, the results obtained by the basic MINLP-based R-DFBA are in accordance with the kinetic modeling results and outperform the remaining approaches; on the other hand, the NLP-based R-DFBA variants predict a constant amount of Ru5P of about 1 mmol l^-1 ^except for the flux-based variant, which also predict a nearly consumed Ru5P after four seconds similar to the kinetic-based results. Surprisingly, the predictions based on the MINLP-based R-DFBA_*CF *_are inconsistent with all other approaches. Here, the minimization of significant fluctuation changes of flux levels and metabolite concentrations results in a short decrease followed by an increase of the Ru5P concentration. We believe that this behavior of the R-DFBA_*CF *_variants is due to the over-constraining of both fluxes and concentrations.

In addition, the PGA concentration closest to the kinetic modeling results is predicted by the MINLP-based R-DFBA approach which minimizes significant flux changes. Contradictory to the results from all other methods, the flux-based R-DFBA, formulated as NLP, predicts an increase of the PGA concentration.

Finally, we analyze the concentration of sink over time. Intriguingly, the sink concentrations are almost constant in the predictions of all NLP-based variants of M-DFBA and R-DFBA. However, for all MINLP-based R-DFBA variants as well as for the DFBA approach, an increase of the sink concentration is observed, which is in line with the kinetic modeling results. Quantitatively the basic MINLP-based R-DFBA approach predicts the best simulation results for the concentration of sink.

Taken together, the results of the RSS-based analysis demonstrate that MINLP-based R-DFBA outperforms M-DFBA in four out of six cases in predicting metabolite concentrations over time.

#### (De)coupling of time-resolved flux predictions

The flux levels over time, also known as time-resolved flux predictions, obtained by the different methods are depicted in Figures S5 - S11 in Additional file 2. By inspecting the results, it becomes apparent that the predictions of the NLP-based variants of both M-DFBA and R-DFBA differ from the outcome of kinetic modeling. The reactions rates are very small for these six methods. Comparing the basic methods with the flux-based variants of M-DFBA and R-DFBA show smoothing of curves, which is also appears in the results of M-DFBA_*CF *_and R-DFBA_*CF*_. Furthermore, it can be observed, that after four seconds for all NLP-based R-DFBA variants the reaction rates are close to zero.

Interestingly, the reaction rates results obtained by DFBA are very close to the kinetic modeling results, as indicated by the RSS values in Table 3. The accuracy of DFBA in the prediction of reaction rates demonstrates that the non-perturbed network may operate towards maximization of sink production. However, we point out that for the reactions v_1_, v_3_, v_4_, v_6 _and v_7 _the predicted reaction rates by DFBA are bounded by the corresponding *v*_*max*_; the latter do not constrain the predictions in the rest of DFBA-based methods.

**Table 3:** Residual sum of squares values for each reaction

Method	Reaction						
	**v**_**1**_	**v**_**2**_	**v**_**3**_	**v**_**4**_	**v**_**5**_	**v**_**6**_	**v**_**7**_
DFBA	5.93	57.44	7.23	6.47	38.49	19.14	0.07
M-DFBA	64.73	175.32	154.96	149.77	76.70	16.70	0.06
Flux-based M-DFBA	58.60	142.74	127.75	131.11	67.76	16.47	0.03
M-DFBA_*CF*_	53.91	143.06	125.41	126.05	67.09	16.85	0.06
R-DFBA NLP	41.13	130.15	113.79	111.48	53.72	16.85	0.06
Flux-based R-DFBA NLP	54.21	187.37	173.83	164.65	82.47	16.30	0.04
R-DFBA_*CF *_NLP	51.68	144.16	143.96	145.26	70.02	16.85	0.06
R-DFBA MINLP	63.95	169.37	158.54	158.91	73.16	17.78	0.06
Flux-based R-DFBA MINLP	47.99	108.16	99.58	102.80	61.20	16.61	0.05
R-DFBA_*CF *_MINLP	25.91	61.38	59.78	60.24	44.04	14.36	0.02

The discrepancy between the kinetic modeling results and the results obtained from the different DFBA-based approaches is quantified based on the residual sum of squares

We argue that to maintain robustness in case of an internal perturbation, the system may opt to optimize additional objectives which are captured by the DFBA-based extensions. Indeed, we observe that the predicted rates obtained by the MINLP-based R-DFBA_*CF *_are closer to the kinetic results than all M-DFBA approaches, as quantified by the RSS.

In the results of the MINLP-based R-DFBA variants, we observe slight local fluctuation which may be attributed to the presence of a large null space in the process of searching an optimal solution of the objective function. A higher-order Legendre polynomial and, therefore, more orthogonal roots in each finite element may reduce these fluctuations. Altogether, these results indicate the decoupling of metabolite concentrations and flux rates in the determined solutions, *i.e.*, that the metabolite concentrations and flux levels change independently of each other. However, it is possible that other optimal solution may reside in the vicinity of the results from the kinetic modeling.

**Figure 4:**
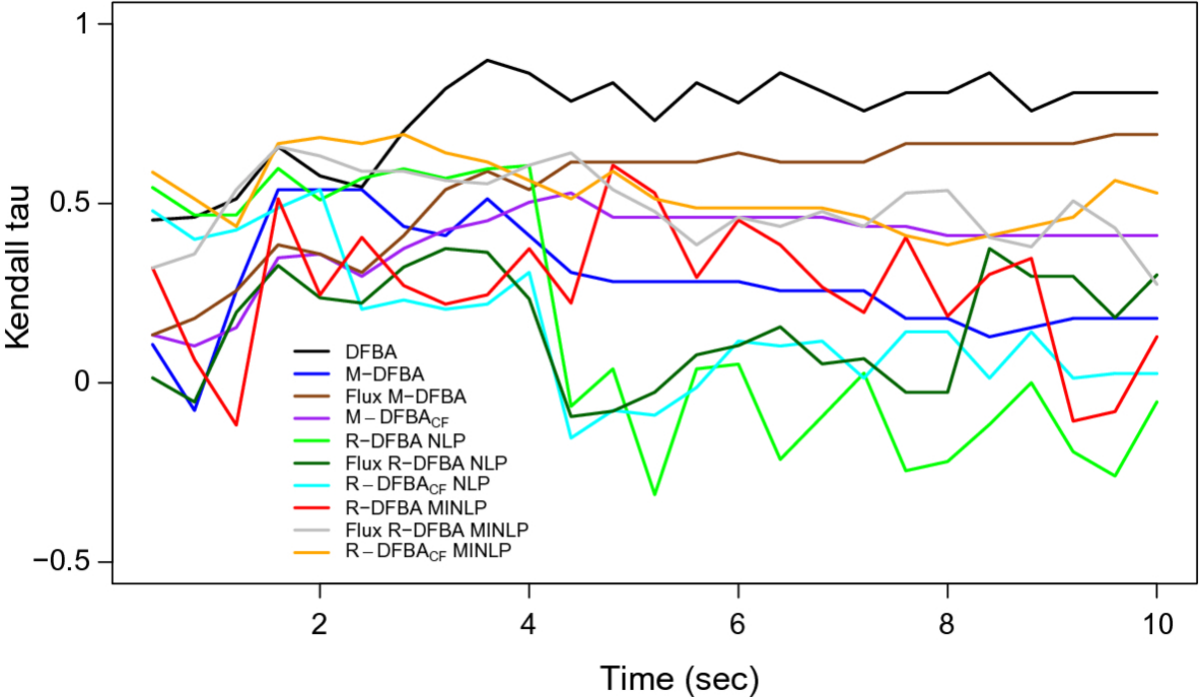
**Time-resolved Kendall correlation coefficient for the outcome of kinetic modeling and each of the constraint-based modeling approaches**. The Kendall correlation coefficient at each time point integrates the vector of metabolite concentrations with that of the flux rates.

To qualitatively examine the extent to which both metabolic concentrations and flux distributions are in agreement with the outcome of kinetic modeling over time, we determine the Kendall τ correlation for the corresponding metabolic states. The results in Figure 4 demonstrate that, qualitatively, the DFBA approach captures best the coupling of flux distributions and metabolic concentrations over time, followed by the flux-based M-DFBA and three of the proposed approaches--the flux-based R-DFBA and R-DFBA_*CF *_based on MINLP as well as M-DFBA_*CF*_.

In addition, these results demonstrate that while the NLP-based R-DFBA methods yield good predictions of metabolite concentrations, the predicted flux distributions over time are decoupled from the metabolite profiles. Interestingly, the same observations for the decoupling of fluxes from metabolite concentrations hold for all M-DFBA-based approaches, except the flux-based M-DFBA. These findings indicate that some results of DFBA-based approaches warrant caution in biological interpretation and applications to metabolic engineering via flux control. Due to the highly coupled regulation of flux and concentrations in metabolic networks [28], it is not surprising that the MINLP-based formulations, especially the flux based R-DFBA and R-DFBA_*CF*_, outperform the rest of the investigated approaches.

### Comparison of the proposed methods and a kinetic model of the plant carbohydrate metabolism

In this section, we analyze the diurnal dynamics of central carbohydrates in leaf cells of wild type *C*_*3*_-plants with the help of the ten implemented methods. In parallel to the wild type, analyses are performed for the *inv4 *mutant, which is defective in the vacuolar invertase gene, At*β*Fruct4 (AT1G12240). Like in the previous section, the performance of the methods to accurately simulate the dynamics of the metabolite concentrations and fluxes is determined based on the RSS values between the results of the constrained-based approaches and the kinetic models for the wild type plant and the *inv4 *mutant (proposed in [9]).

The models for both, wild type and mutant, include six metabolites, namely: glucose, fructose, sucrose, sugar phosphates, and starch, as well as a combined sink export, which are interconverted through seven reactions (Figure S12 in Additional file 3). These models include the most abundant sugars and sugar phosphates in the carbon metabolism of plants, without resolving subcellular compartments. Therefore, they sacrifice the complexity to increase feasibility of simulation studies. The reactions are modeled over a 24 h diurnal cycle with inclusion of limited knowledge regarding the kinetic parameters in the models of the wild type and the mutant. Therefore, these examples demonstrate that the constrained-based formulations proposed in this study support also the inclusion of kinetic information. The kinetic models used in these comparisons depend on publicly unavailable measurements. However, among others, the rate of net photosynthesis was approximated by a smoothing spline interpolation of measurements over whole diurnal cycles. Furthermore, unknown model parameters were identified by minimizing the sum of squared errors between simulated and measured states [9]. As a result, these case scenarios of comparing the performances with the results from the kinetic models can be viewed as a proxy for the data-driven comparison.

The simulation day begins at 6 o'clock and has a 16 hours light and 8 hours dark phase. The time period is divided into twelve finite elements, and orthogonal collocation is applied to a time interval of two hours. This results in at least 780 variables which need to be optimized based on over 1000 constraints, depending on the different approaches. For the DFBA approach, maximal sinks production is supposed to be the systemic objective to be optimized.

#### RSS for metabolite concentrations and reaction fluxes

Inspection of the RSS results for the metabolite concentrations, presented in Table S1 and S3 in Additional file 3 for the wild type and the *inv4 *mutant, respectively, support the statements made in the previous section, dealing with the model of the Calvin-Benson cycle-the NLP-based R-DFBA_*CF *_variant outperforms not only the three M-DFBA variants, but also the classical DFBA based on maximal sink production for the wild type as well as the mutant. In addition, the same claim holds for majority of the metabolites for the basic and the flux-based NLP variants of R-DFBA.

With respect to the reaction fluxes for the wild type as well as for the *inv4 *mutant, the flux-based M-DFBA outperforms all other methods; nevertheless, the basic NLP-based R-DFBA ranks second. Inspection of the RSS results, presented in Table S2 and S4 in Additional file 3, are in line with our observation that constraint-based approaches result in decoupling of the metabolite concentrations and reaction fluxes. Nevertheless, taken together, these findings indicate that the proposed constraint-based approaches for simulating the dynamics of metabolic networks may be a suitable tool in analyzing models of metabolic processes for which little information, aside from stoichiometry, is available.

### Challenges in comparison of experimental data with the findings from method applications

The time-dependent concentration and flux profiles predicted by the proposed methods are in good agreement with the respective profiles generated from the kinetic models of the Calvin-Benson cycle and carbohydrate metabolism. Nevertheless, the ultimate biological validity of the minimization principle(s), used to obtain the aforementioned predications, remains to be established with the help of experimental data of in vivo metabolite concentrations and intracellular reaction fluxes. However, designing and conducting experiments from which non-stationary metabolite concentrations and reaction fluxes are to be measured is an extremely challenging task. For instance, decades of experimental work for quantifying the absolute concentrations of photosynthetic intermediates [42] and end-product pathways have resulted in methods applicable for obtaining the in vivo steady-state levels for 40 metabolites [39]. Moreover, virtually all existing methods for determining intracellular fluxes rely on model estimates generated with the help of labeled metabolomics data and the steady-state assumption [43-45]. Finally, the methods for monitoring the non-stationary state are currently not suited for system-level probing. Given the state-of-the-art, we employed synthetic data, generated from the considered models, as a first step in validating the claims.

### Application to genome-scale metabolic networks

The analysis of genome-scale networks is the focus of current modeling strategies with many different applications, including metabolic engineering and drug targeting [46]. Furthermore, in the last years, the quality of genome-scale models has improved due to the incorporation of extensive experimental data, resulting in a large number of included reactions and metabolites. For instance, the new reconstruction of *Escherichia coli *includes 2,251 metabolic reactions, and 1,136 unique metabolites [47]. An accurately simulation of the behavior of this network over 10 seconds using DFBA-based methods with the same setting as for the Calvin-Benson cycle model, would result in at least 56,275 unknown variables for the flux rates and 28,400 for the metabolite concentrations over time. It is important to note, that while solving some special classes of NLP can be performed in polynomial time, MINLP problems are often not tractable (*i.e.*, they belong to the class of NP-hard problems [33,48]). Therefore, an application of DFBA-based methods to genome-scale models is currently hampered by the size of the resulting instances as well as the lack of optimization platforms which scale well. In addition, a comparison of the predictive power of the different methods is only possible by including experimental data, or at least kinetic model predictions, as it is the case for our analysis. To our knowledge, kinetic model based predictions, and particularly experimental data of the profile of metabolite concentrations and flux levels, are not available for genome-scale models. Nevertheless, as shown above, the predictive power demonstrates that DFBA-based methods are useful for an accurate prediction of time-resolved metabolite concentrations and flux rates of metabolic networks for which kinetic parameters cannot be obtained but solving at least the NLP problems are possible.

## Conclusions

In the present work, we proposed a new approach to analyze the dynamic adjustment of metabolic networks, called R-DFBA. R-DFBA combines the dynamic FBA with regulatory on/off minimization by minimizing the total number of significant metabolite concentration changes in comparison to flux changes in the classical approach. Additionally, we extended this method and the M-DFBA approach by considering not only the fluctuation of the metabolite concentration profiles but also those of the flux levels in the network. We introduced seven new approaches and, in total, ten constraint-based approaches were implemented and their accuracy was compared with the outcome of two kinetic models--for the Calvin-Benson cycle and for the plant carbohydrate metabolism.

For the models of the Calvin-Benson cycle and the plant carbohydrate metabolism, we demonstrated that our proposed approaches yielded results in accordance with predictions from kinetic modeling, specifically for the case of metabolite concentrations. In addition, we demonstrated that the (MI)NLP-based R-DFBA approach captures the dynamic coupling of reaction fluxes and metabolite concentrations. Therefore, constraint-based approaches in combination with collocation on finite elements offer a promising framework for analysis of metabolic network dynamics without specifying the details of enzyme kinetics. The proposed approaches outperform the existing variants in several cases and are suitable for positing model-based hypotheses for the dynamics of metabolic pathways when little enzymatic details are available. Finally, our findings suggest that minimizing the combination of flux and metabolite concentration fluctuations is the mechanism most likely responsible for maintaining the metabolic network robustness due to internal perturbations.

## Methods

### Statistical analysis

The results of applying the described methods are compared with the outcome from kinetic modeling with the help of residual sum of squares (RSS). The RSS for each system element (*i.e.*, metabolite and reaction flux) is defined as follows:

$$RSS=\overset{M}{\underset{j=1}{\sum }}{\left(y_{j}-f\left(x_{j}\right)\right)}^{2},$$

where *y*_*j *_is the result of the kinetic modeling and *f*(*x*_*j*_) of the compared approaches at the orthogonal root *j*.

The Kendall rank correlation coefficient, denoted by τ, evaluates the degree of similarity between two sets of ranks given to a same set of objects [49]. This coefficient depends upon the number of inversions of pairs of objects which would be needed to transform one rank order into the other. We use the Kendall τ to qualitatively discriminate between the constraint-based approaches with respect to their correspondence to the outcome of kinetic modeling by using the temporal distribution of both metabolic concentrations and reaction fluxes.

### Implementation

All mathematical programming approaches are implemented in MATLAB 7.11.0, R2010b with the optimization platform TOMLAB v7.7 [50]. We use CPLEX to solve LP problems, the SNOPT solver for NLP problems and MINLPbb for MINLP problems. The kinetic modeling of the Calvin-Benson cycle is performed by using the biochemical network simulator COPASI 4.6 [51], while the kinetic modeling of the plant carbohydrate metabolism is conducted with the Systems Biology Toolbox2 [52].

## Authors' contributions

SK and ZN designed the study and developed the mathematical formulation of the methods. SK carried out the implementation and computational analysis. SK and ZN performed the statistical and comparative analyses, and were both involved in writing the manuscript. All authors read and approved the final manuscript.

## Supplementary Material

Additional file 1 **Tutorial Orthogonal Collocation on Finite Elements**. Tutorial for the orthogonal collocation on finite elements is included. An illustrative example demonstrating the usage of this method in approximating solutions to ODEs is also presented. Click here for file

Additional file 2 **Additional Figures**. Modeling results of concentrations of Ru5P, PGA, GAP and sink as well as results of reaction rates of v_1_-v_7 _in the simplified model of the Calvin cycle by the different approaches. Click here for file

Additional file 3 **Additional results Photosynthesis Model**. Results from the RSS analysis of the proposed methods for a lumped model of C_3_-plant carbohydrate metabolism are presented. The model includes 6 metabolites and 7 reactions, as depicted in Figure S12. In addition, the ODEs for the kinetic model used in establishing the RSS results are presented. Click here for file
