# Supplementary material for: Dynamic regulatory on/off minimization for biological systems under internal temporal perturbations
Source: BMC Syst Biol. 2012 Mar 12;6:16. doi: 10.1186/1752-0509-6-16 (PMC3361480; doi:10.1186/1752-0509-6-16)
Supplement: Additional file 1 — Tutorial Orthogonal Collocation on Finite Elements. Tutorial for the orthogonal collocation on finite elements is included. An illustrative example demonstrating the usage of this method in approximating solutions to ODEs is also presented. [file 1752-0509-6-16-S1.PDF]

Tutorial

Orthogonal Collocation on Finite Elements

Sabrina Kleessen[1] and Zoran Nikoloski[1,2]

1. Max-Planck Institute of Molecular Plant Physiology, Potsdam, Germany
2. Institute of Biochemistry and Biology, University of Potsdam, Potsdam, Germany

Consider a simple metabolic network consisting of three reactions involving four metabolites A, B, C and E:

$$\begin{aligned} C &\xrightarrow{v_1} A + B \\ 2E &\xrightarrow{v_2} B + C \\ O &\xrightarrow{v_3} E, \end{aligned}$$

where O denotes the environment.
For this reaction network the stoichiometric matrix is given by:

$$S = \begin{pmatrix} 1 & 0 & 0 \\ 1 & 1 & 0 \\ -1 & 1 & 0 \\ 0 & -2 & 1 \end{pmatrix},$$

where rows correspond to metabolites and columns to reactions, respectively.
Thus, the differential equations of the network are given by:

$$\begin{aligned} \frac{dA}{dt} &= v_1, \\ \frac{dB}{dt} &= v_1 + v_2, \\ \frac{dC}{dt} &= -v_1 + v_2, \\ \frac{dE}{dt} &= -2v_2 + v_3, \end{aligned}$$

which represent changes in metabolite levels in terms of the reaction rates v_m, $1 \leq m \leq 3$.

A general optimization problem including a dynamic model described by differential equations can be posed as:

$$
\begin{aligned}
\text{Objective :} \quad & \max f(t) \quad t \in [t_0, t_f] \\
s.t. \quad & \\
& \tfrac{du}{dt} = F[u(t), z(t), t] \\
& u(t_0) = u_0 \\
& u(t)^L \leq u(t) \leq u(t)^U \\
& z(t)^L \leq z(t) \leq z(t)^U,
\end{aligned} \tag{1}
$$

where $f(t)$ is an objective function, $u(t)$ denotes the state profile vector, and $z(t)$ is a control profile vector, while u_0 give the initial conditions for the state profile vector. The bounds of the state profile vector are $u(t)^L, u(t)^U$ and the bounds of the control profile vector are $z(t)^L, z(t)^U$.
A linear optimization problem that includes the differential equations for the four metabolites A, B, C and E is then given by:

$$
\begin{aligned}
\text{Objective :} \quad & \max f(t) \quad t \in [t_0 = 0, t_f = 3] \\
s.t. \quad & \\
& \tfrac{dA}{dt} = v_1 \\
& \tfrac{dB}{dt} = v_1 + v_2 \\
& \tfrac{dC}{dt} = -v_1 + v_2 \\
& \tfrac{dE}{dt} = -2v_2 + v_3 \\
& A(t_0) = A_0 = 2 \\
& B(t_0) = B_0 = 2 \\
& C(t_0) = C_0 = 2 \\
& E(t_0) = E_0 = 2 \\
& 0 = A(t)^L \leq A(t) \leq A(t)^U = 100 \\
& 0 = B(t)^L \leq B(t) \leq B(t)^U = 100 \\
& 0 = C(t)^L \leq C(t) \leq C(t)^U = 100 \\
& 0 = E(t)^L \leq E(t) \leq E(t)^U = 100 \\
& 0 = v_m(t)^L \leq v_m(t) \leq v_m(t)^U = 100 \quad m = 1, \ldots, 3,
\end{aligned} \tag{2}
$$

where v_m are the flux rates of the reactions. In case of the simple metabolic network, $v_m(t)$ are the components of the control profile vectors and $A(t)$, $B(t)$, $C(t)$ and $E(t)$ are the components of the state profile vectors. Moreover, as an objective function, the maximization the sum over the concentration of metabolite E at different time points ($f(t) = \sum E(t)$) can be used.

Collocation method

An optimization problem including differential equations cannot be solved directly. Before solving the problem a solution for the differential equations needs to be determined. Therefore, the differential equations can be solved by different approximation techniques. In our case, we use the orthogonal collocation method to approximate the exact solution for the system of ordinary differential equations (ODEs) [1]. Suppose we have a differential equation, with a differential operator D acting on a function u:

$$D(u(t)) = \frac{du}{dt}, \qquad t \in [t_0, t_f], \tag{3}$$

with $u(t_0) = u_0$. Hence, the differential equation of metabolite A can be described by:

$$D(A(t)) = \frac{dA}{dt}, \qquad t \in [t_0, t_f], \tag{4}$$

with $A(t_0) = A_0$, where A_0 represents the initial concentration of metabolite A at time point t_0. In general we are interested in the function u, which we want to approximate by $\tilde{u}$, where $\tilde{u}$ is a combination of polynomials:

$$u(t) \cong \tilde{u}(t) = \sum_{j=0}^{K} a_j \cdot \phi_j(t). \tag{5}$$

As polynomials, one most commonly uses the family of Lagrange polynomials whereby

$$\phi_j(t) = \prod_{\substack{0 \le k \le K \\ k \ne j}} \frac{t - t_k}{t_j - t_k}. \tag{6}$$

In the majority of cases, the polynomial given in Eq. (5) can only be used for the state profile vectors due to missing initial conditions for the control profile vectors $z(t)$. Thus, the approximation by polynomials for $z(t)$ is defined as follows:

$$z(t) \cong \tilde{z}(t) = \sum_{j=1}^{K} b_j \cdot \psi_j(t); \qquad \psi_j(t) = \prod_{\substack{1 \le k \le K \\ k \ne j}} \frac{t - t_k}{t_j - t_k}. \tag{7}$$

The result of the differentiation of $\tilde{u}(t)$ is not, in general $\frac{du}{dt}$. This yields to a residual equation [2]:

$$R(t) = \frac{d\tilde{u}(t)}{dt} - \frac{du(t)}{dt}. \tag{8}$$

For our example the residual equation of metabolite A is given by:

$$R_A(t) = \frac{d\tilde{A}(t)}{dt} - \frac{dA(t)}{dt}, \tag{9}$$

where $\tilde{A}(t)$ is the description of the unknown exact solution for $A(t)$ by the assumed polynomials. The aim of collocation methods is to force the results of $R(t)$ to zero. Then, the discretization of the residual equation based on the method of collocation is performed as follows:

$$\int_{t_0}^{t_f} R(t)\delta(t-t_i)dt = 0, \qquad i = 1, \ldots, K, \tag{10}$$

where δ represents the Dirac delta function [3] and t_0 and t_f are the bounds of the interval (time period). The Dirac delta function has the property that it is zero everywhere except at the origin, where it is infinite:

$$\delta(x) = \begin{cases} +\infty, & \text{if } x = 0 \\ 0, & \text{if } x \neq 0. \end{cases} \tag{11}$$

A schematic representation of the function is given in Figure 1. The Dirac delta

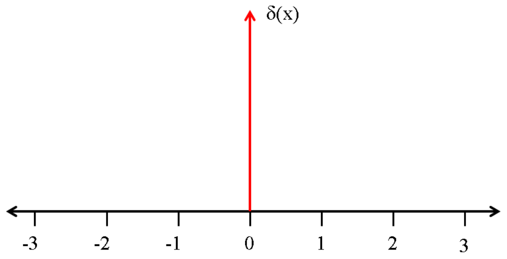

Figure 1: Schematic representation of the Dirac delta function.

function is not a true function, formally it can be defined as a distribution. At $x_0 = 0$ the distribution is infinity, consequently the result is a finite integral:

$$\int_{-\infty}^{\infty} \delta(x)dx = 1. \tag{12}$$

For an integrable function $f(x)$ we have that

$$\int_{-\infty}^{\infty} f(x)\delta(x)dx = f(0), \tag{13}$$

which states that the integral of any function multiplied by the Dirac delta function is just the value of the function at zero. If the Dirac Delta function is shifted to $x = x_0$ by definition of $\delta(x - x_0)$ the result is just the value of the function f at x_0:

$$\int_{-\infty}^{\infty} f(x)\delta(x-x_0)dx = f(x_0). \tag{14}$$

Accordingly, the integral over the residual function $R(t)$ can be written as:

$$R(t_i) = \frac{d\tilde{u}(t_i)}{dt} - \frac{du(t_i)}{dt} = \sum_{j=0}^{K} a_j \cdot \frac{d\phi_j(t_i)}{dt} - \frac{du(t_i)}{dt}, \; i = 1, \ldots, K, \tag{15}$$

where t_i are discrete points.
The evaluation of the polynomials at discrete points t_i, $t_0 < t_i < t_f$, reduces to the coefficients a_j at these points due to the Lagrange condition, defined by $u_j(t_i) = \delta_{ji}$, where in this case δ_{ji} is the Kronecker delta. The Kronecker delta is a function of two variables, defined as follows:

$$\delta_{ji} = \begin{cases} 1, & \text{if } j = i \\ 0, & \text{if } j \neq i. \end{cases} \tag{16}$$

Therefore, only a solution for the unknown coefficients a_j needs to found. The location of the points t_i, $i = 1, ...K$, named *collocation points*, are chosen to correspond to the shifted roots of an orthogonal Legendre polynomial of degree K [1]. Note that Legendre polynomials are solutions to the Legendre's differential equation:

$$\frac{d}{dx}\left[(1-x^2)\frac{d}{dx}P_n(x)\right] + n(n+1)P_n(x) = 0. \tag{17}$$

For example the fifth order Legendre polynomial is defined by:

$$P_5(x) = \frac{1}{8}(63x^5 - 70x^3 + 15x), \tag{18}$$

which obviously results in five (orthogonal) roots. For a time period of $t_0 = 0$ and $t_f = 1$ the roots are shown in Figure 2.

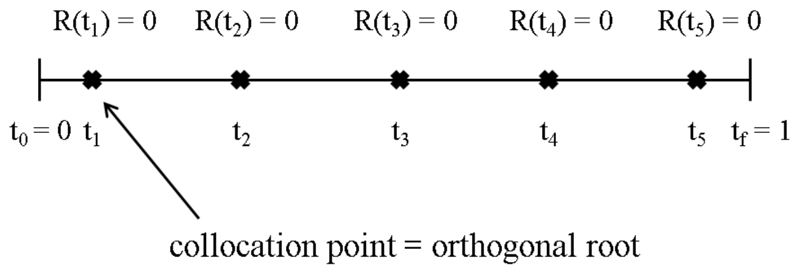

Figure 2: Orthogonal roots of the fifth order Legendre polynomial in the interval $[t_0 = 0, t_f = 1]$, where $t_1 = 0.0469101$, $t_2 = 0.23076535$, $t_3 = 0.5$, $t_4 = 0.76923465$ and $t_5 = 0.9530899$.

Parameterizing the dynamic equations of the metabolites A, B, C and E results in the representation of the seven variables (for metabolite concentrations and flux rates) by novel parameters at each orthogonal root, depicted in Figure 3.

Therefore, the equations for metabolite A considering $K = 5$ at the orthogonal roots are given by:

$$A(t_i) = A_0 \cdot \phi_0(t_i) + A_1 \cdot \phi_1(t_i) + A_2 \cdot \phi_2(t_i) + A_3 \cdot \phi_3(t_i) + A_4 \cdot \phi_4(t_i) + A_5 \cdot \phi_5(t_i), \quad i = 1, \ldots, 5. \quad (19)$$

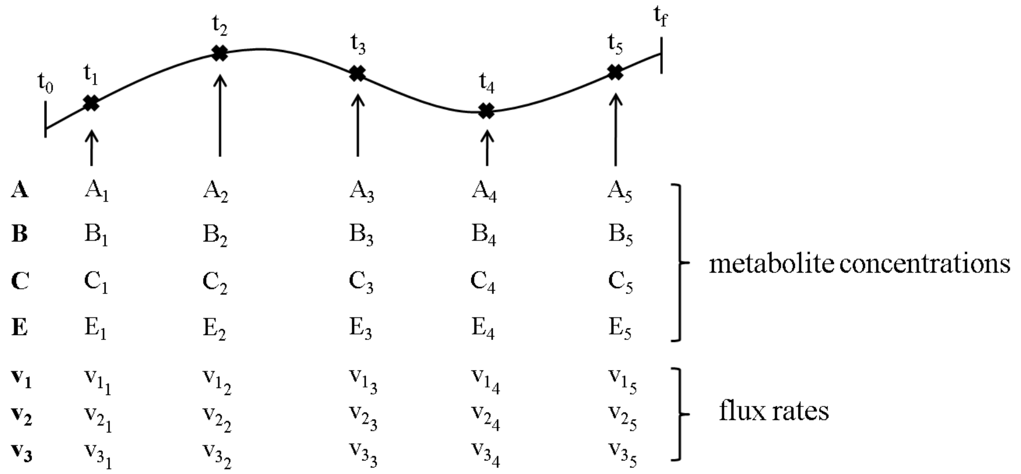

Figure 3: Representation of the variables by new parameters at discrete points t_1 to t_5.

Hence, the equation $\frac{dA}{dt} = v_1$ is parameterized at the roots of the orthogonal polynomial by the following:

$$A_0 \cdot \dot{\phi}_0(t_i) + A_1 \cdot \dot{\phi}_1(t_i) + A_2 \cdot \dot{\phi}_2(t_i) + A_3 \cdot \dot{\phi}_3(t_i) + A_4 \cdot \dot{\phi}_4(t_i) + A_5 \cdot \dot{\phi}_5(t_i) - (v_{1_i}) = 0, \quad i = 1, \ldots, 5, \quad (20)$$

with

$$\dot{\phi}_j(t_i) = \frac{d\phi_j}{dt}, \quad j = 0, \ldots, 5. \quad (21)$$

The equations of the metabolites B, C and E can be parameterized analogously.

Extension to orthogonal collocation on finite elements

Above we describe the global collocation. The global collocation requires a very large number of coefficients for an acceptable approximation of functions that contain both steep fronts and flat regions (requiring a very large K). An alternative to global collocation uses piecewise polynomial approximations. For the extension from the global (orthogonal) collocation to orthogonal collocation on finite elements, the interval (time period) is divided into a number of intervals, named *finite elements* [4, 5, 6]. Orthogonal collocation is then applied to each finite element. A finite element $\Delta\zeta_n$ with $n = 1, \ldots, e$, where e is the number of finite elements, is bounded by two points ζ_n and ζ_{n+1} with $\Delta\zeta_n = \zeta_n - \zeta_{n+1}$. The

orthogonal properties obtained with global collocation are preserved by mapping the interval $t \in [t_0, t_f]$ used in the global collocation into each finite element $\Delta\zeta_n$ by $\zeta_n = t_0$ and $\zeta_{n+1} = t_f$. Thus, the location of the Legendre polynomials are mapped to the points:

$$t_{n,i} = \zeta_n + t_i, \qquad n = 1, \dots, e \qquad i = 0, \dots, K. \tag{22}$$

In Figure 4, $K = 5$ and the number of finite elements is three ($e = 3$). Accordingly, the orthogonal roots of $\Delta\zeta_2$ from the example in Figure 4 are given by $t_{2,1} = 1.0469101$, $t_{2,2} = 1.23076535$, $t_{2,3} = 1.5$, $t_{2,4} = 1.76923465$ and $t_{2,5} = 1.9530899$.

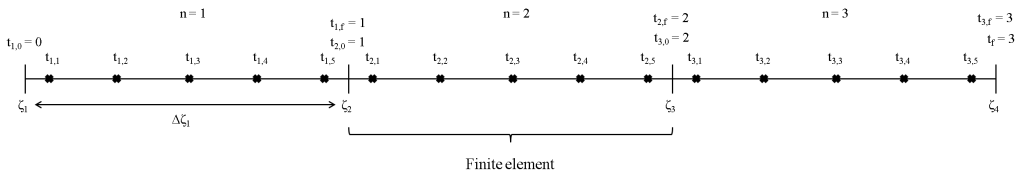

Figure 4: Illustration of the evaluation points in an example with $K = 5$ and $e = 3$. Considered are the time period $[t_0 = 0, t_f = 3]$.

With help of the orthogonal collocation on finite elements the solution of the differential equations can now be approximated by:

$$\tilde{u}(t) = \sum_{j=0}^{K} a_{n,j} \cdot \phi_j(t); \qquad \phi_j(t) = \prod_{\substack{0 \leq k \leq K \\ k \neq j}} \frac{t - t_{n,k}}{t_{n,j} - t_{n,k}}, \qquad n = 1, \dots, e. \tag{23}$$

To enforce continuity at the endpoints of each finite element the polynomial $\tilde{u}(t)$ is extrapolated to $t_{n,f}$ with

$$\tilde{u}(\zeta_{n+1}) = \tilde{u}(t_{n,f}) = \sum_{j=0}^{K} a_{n,j} \cdot \phi_j(t_{n+1,0}) = \sum_{j=0}^{K} a_{n,j} \cdot \phi_j(t_{n,f}), \quad n = 1, \dots, e-1. \tag{24}$$

The result of the extrapolation provides an accurate initial condition $a_{n+1,0}$ for the next finite element and polynomial $\tilde{u}(\zeta_{n+1})$.
The optimization problem given in Eq. (1) can now be solved easily with any solver. For the example metabolic network, the linear programming (LP) formulation described in Eq. (2) including the ODEs discretized on finite elements and the

continuity conditions becomes:

$$
\begin{aligned}
&\text{Objective}: \quad \max \sum E(t_{n,i}) \quad n = 1, \ldots, 3 \;\; i = 1, \ldots, 5\\
&s.t.\\
&A(t_{1,0}) = A_0 = 2\\
&B(t_{1,0}) = B_0 = 2\\
&C(t_{1,0}) = C_0 = 2\\
&E(t_{1,0}) = E_0 = 2\\
&0 = A(t)^L \le A(t) \le A(t)^U = 100\\
&0 = B(t)^L \le B(t) \le B(t)^U = 100\\
&0 = C(t)^L \le C(t) \le C(t)^U = 100\\
&0 = E(t)^L \le E(t) \le E(t)^U = 100\\
&0 = v_m(t)^L \le v_m(t) \le v_m(t)^U = 100 \quad m = 1, \ldots, 3,\\
&\forall n, i \;\; 1 \le n \le 3, \; 1 \le i \le 5 \qquad (25)\\
&R_A(t_{n,i}) = \tfrac{d\tilde{A}(t_{n,i})}{dt} - \tfrac{dA(t_{n,i})}{dt} = \textstyle\sum_{j=1}^{K} A_{n,j} \cdot \dot{\phi}_j(t_{n,i}) - (v_{1_{n,i}}) = 0\\
&R_B(t_{n,i}) = \tfrac{d\tilde{B}(t_{n,i})}{dt} - \tfrac{dB(t_{n,i})}{dt} = \textstyle\sum_{j=1}^{K} B_{n,j} \cdot \dot{\phi}_j(t_{n,i}) - (v_{1_{n,i}} + v_{2_{n,i}}) = 0\\
&R_C(t_{n,i}) = \tfrac{d\tilde{C}(t_{n,i})}{dt} - \tfrac{dC(t_{n,i})}{dt} = \textstyle\sum_{j=1}^{K} C_{n,j} \cdot \dot{\phi}_j(t_{n,i}) - (-v_{1_{n,i}} + v_{2_{n,i}}) = 0\\
&R_E(t_{n,i}) = \tfrac{d\tilde{E}(t_{n,i})}{dt} - \tfrac{dE(t_{n,i})}{dt} = \textstyle\sum_{j=1}^{K} E_{n,j} \cdot \dot{\phi}_j(t_{n,i}) - (-2v_{2_{n,i}} + v_{3_{n,i}}) = 0\\
&\forall n \;\; n = 1\\
&\textstyle\sum_{j=1}^{K} A_{n,j} \cdot \phi_j(0) - A_0 = 0\\
&\textstyle\sum_{j=1}^{K} B_{n,j} \cdot \phi_j(0) - B_0 = 0\\
&\textstyle\sum_{j=1}^{K} C_{n,j} \cdot \phi_j(0) - C_0 = 0\\
&\textstyle\sum_{j=1}^{K} E_{n,j} \cdot \phi_j(0) - E_0 = 0\\
&\forall n \;\; 2 \le n \le 3\\
&\textstyle\sum_{j=1}^{K} A_{n-1,j} \cdot \phi_j(1) - \sum_{j=1}^{K} A_{n,j} \cdot \phi_j(0) = 0\\
&\textstyle\sum_{j=1}^{K} B_{n-1,j} \cdot \phi_j(1) - \sum_{j=1}^{K} B_{n,j} \cdot \phi_j(0) = 0\\
&\textstyle\sum_{j=1}^{K} C_{n-1,j} \cdot \phi_j(1) - \sum_{j=1}^{K} C_{n,j} \cdot \phi_j(0) = 0\\
&\textstyle\sum_{j=1}^{K} E_{n-1,j} \cdot \phi_j(1) - \sum_{j=1}^{K} E_{n,j} \cdot \phi_j(0) = 0,
\end{aligned}
$$

where $A_{n,i}$, $B_{n,i}$, $C_{n,i}$ and $E_{n,i}$ are the concentrations of each metabolite and $v_{1_{n,i}}$, $v_{2_{n,i}}$ and $v_{3_{n,i}}$ are the reaction rates at time points (collocation points) $t_{n,i}$, $1 \le n \le 3$, $1 \le i \le 5$, respectively. The solution of the coefficients at the orthogonal roots are shown in Table 1.

Coefficients at	Metabolites				Reactions		
orthogonal roots	A	B	C	E	v_1	v_2	v_3
$t_{1,1}$	2.1718	2.1699	1.8264	6.6948	3.5145	0.0000	100.0000
$t_{1,2}$	2.7041	2.7083	1.3000	25.0679	2.2731	0.0000	100.0000
$t_{1,3}$	3.1272	3.1148	0.8604	52.0258	1.1273	0.0000	100.0000
$t_{1,4}$	3.5343	3.6886	0.6199	78.6026	2.4683	1.6725	99.8657
$t_{1,5}$	4.2570	4.9705	0.4566	95.8246	5.7828	4.7368	99.6196
$t_{2,1}$	5.3734	8.2838	1.5370	100.0000	17.0817	40.9647	81.9294
$t_{2,2}$	8.1914	18.1922	5.8095	100.0000	13.3301	36.1209	72.2418
$t_{2,3}$	11.0149	29.8208	11.7909	100.0000	8.2626	29.5769	59.1538
$t_{2,4}$	13.3374	39.5453	16.8704	100.0000	10.9606	26.1612	52.3224
$t_{2,5}$	16.1823	47.2328	18.8681	100.0000	21.4360	27.0487	54.0973
$t_{3,1}$	18.3737	52.0302	19.2827	100.0000	21.8114	27.4171	54.8342
$t_{3,2}$	21.5117	59.7985	20.7750	100.0000	13.0969	23.4084	46.8168
$t_{3,3}$	24.3361	68.6359	23.9638	100.0000	10.0392	22.0947	44.1893
$t_{3,4}$	28.2376	78.8247	26.3496	100.0000	21.6905	25.2728	50.5456
$t_{3,5}$	33.7625	89.3620	25.8370	100.0000	39.9244	29.4936	58.9871

Table 1: Results of metabolite concentrations and reaction rates at different orthogonal roots. The LP problem given in Eq. (25) was solved by the linprog function in MATLAB.

References

[1] Villadsen JV, Stewart WE: **Solution of boundary-value problems by orthogonal collocation**. *Chemical Engineering Science* 1995, **50**(24):3981–3996.

[2] Villadsen J, Michelsen ML: *Solution of differential equation models by polynomial approximation.* Prentice Hall, Englewood Cliffs, New Jersey edition 1978.

[3] Dirac P: *Principles of quantum mechanics*, Oxford at the Clarendon Press. 4 edition 1958 :58.

[4] Carey GF, Finlayson BA: **Orthogonal collocation on finite elements**. *Chemical Engineering Science* 1975, **30**:587–596.

[5] Cuthrell JE, Biegler LT: **On the optimization of differential-algebraic process systems**. *American Institute of Chemical Engineers Journal* 1987, **33**(8):1257–1270.

[6] Čižniar M, Salhi D, Fikar M, Latifi M: **A MATLAB package for orthogonal collocations on finite elements in dynamic optimization**. *15th Int. Conference Process Control, June 7-10, 2005, Štrbské Pleso, Slovakia* 2005.
