# Supplementary material for: Dynamic regulatory on/off minimization for biological systems under internal temporal perturbations
Source: BMC Syst Biol. 2012 Mar 12;6:16. doi: 10.1186/1752-0509-6-16 (PMC3361480; doi:10.1186/1752-0509-6-16)
Supplement: Additional file 2 — Additional Figures. Modeling results of concentrations of Ru5P, PGA, GAP and sink as well as results of reaction rates of v1-v7 in the simplified model of the Calvin cycle by the different approaches. [file 1752-0509-6-16-S2.PDF]

Additional figures

Figure S1

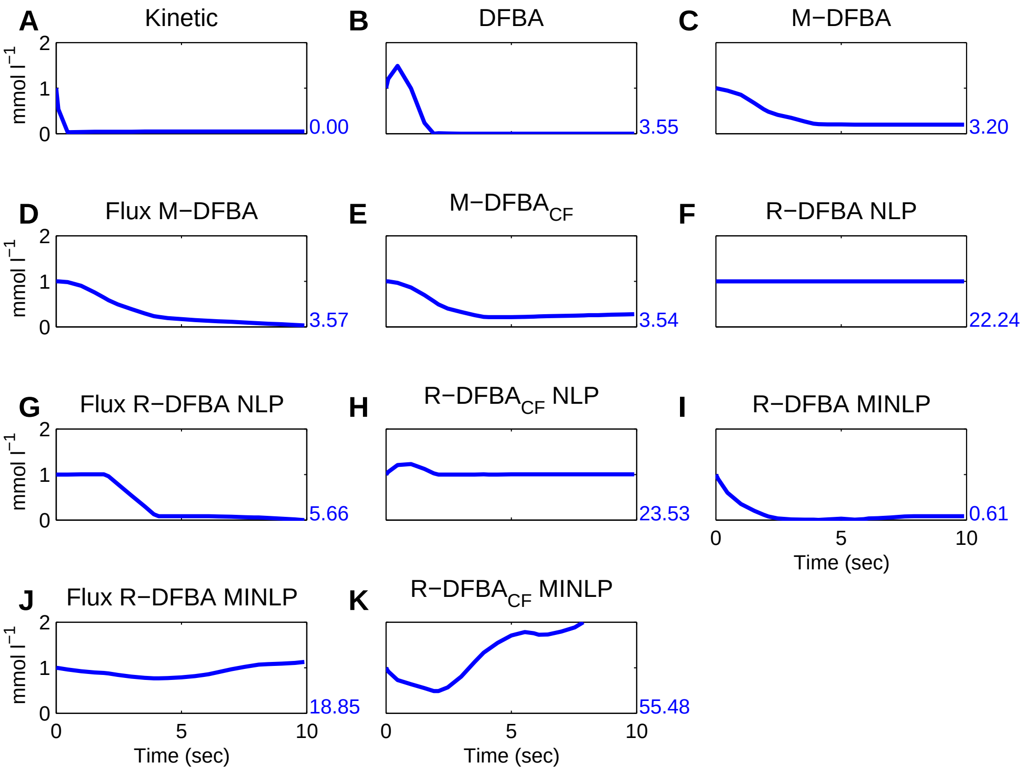

Figure S1: Modeling results of concentrations of **Ru5P** in the simplified model of the Calvin cycle by the different approaches. The residual sum of squares value of each approach is presented in the bottom right of the corresponding subfigure.

Figure S2

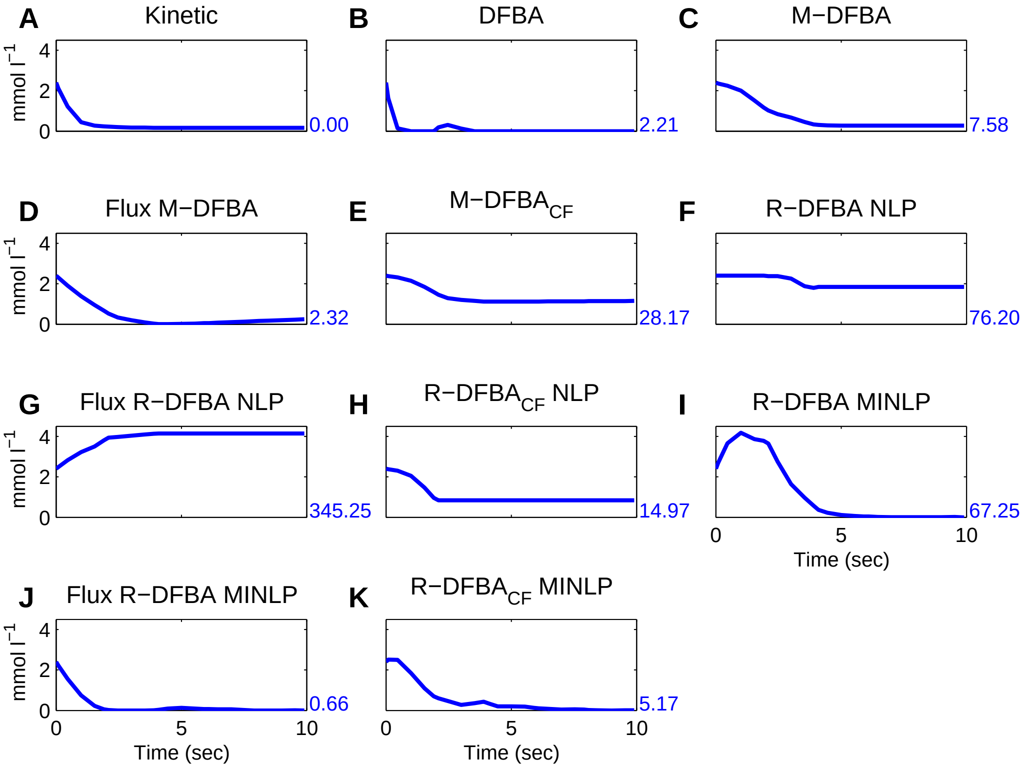

Figure S2: Modeling results of concentrations of **PGA** in the simplified model of the Calvin cycle by the different approaches. The residual sum of squares value of each approach is presented in the bottom right of the corresponding subfigure.

Figure S3

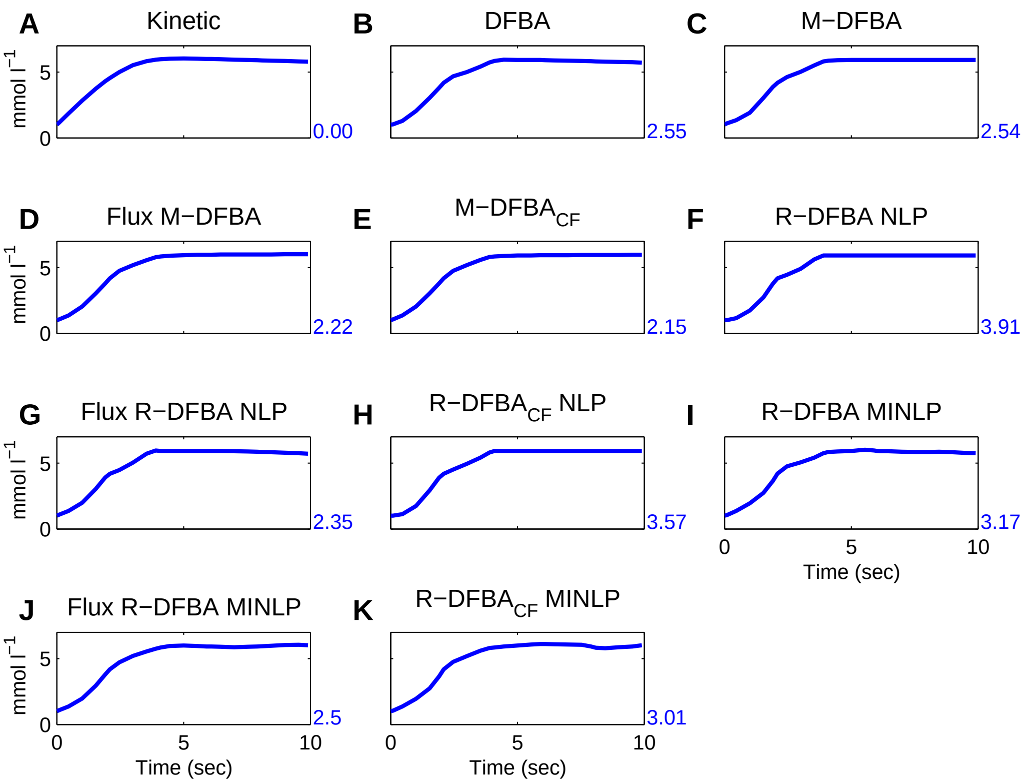

Figure S3: Modeling results of concentrations of **GAP** in the simplified model of the Calvin cycle by the different approaches. The residual sum of squares value of each approach is presented in the bottom right of the corresponding subfigure.

Figure S4

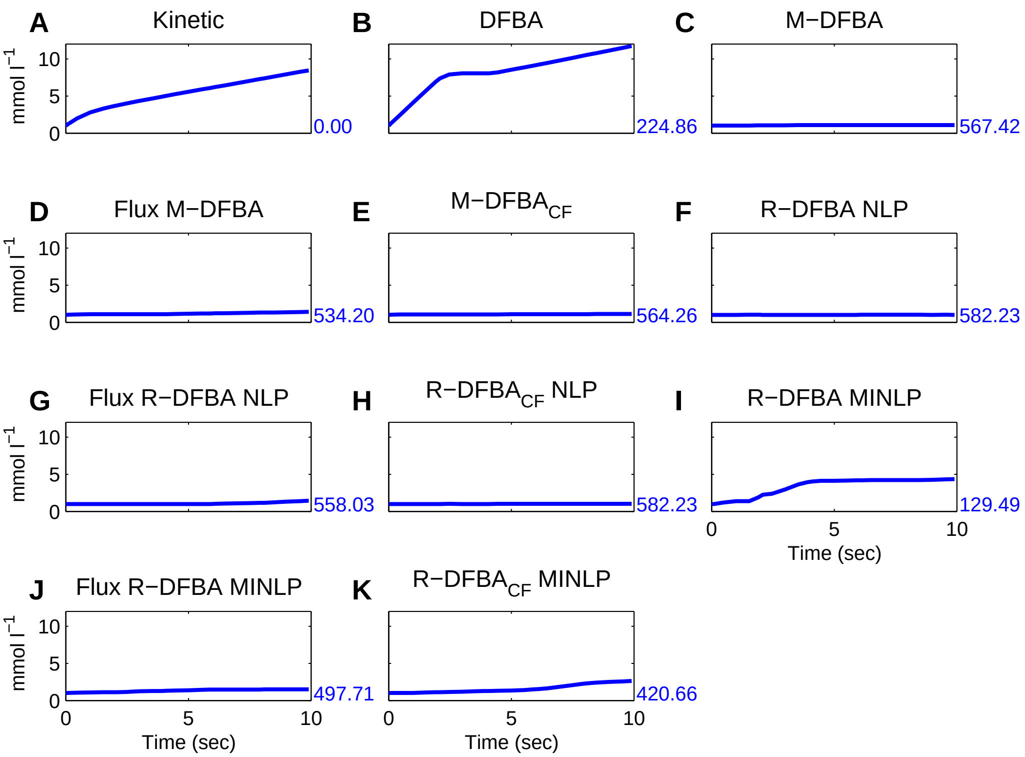

Figure S4: Modeling results of concentrations of **sink** in the simplified model of the Calvin cycle by the different approaches. The residual sum of squares value of each approach is presented in the bottom right of the corresponding subfigure.

Figure S5

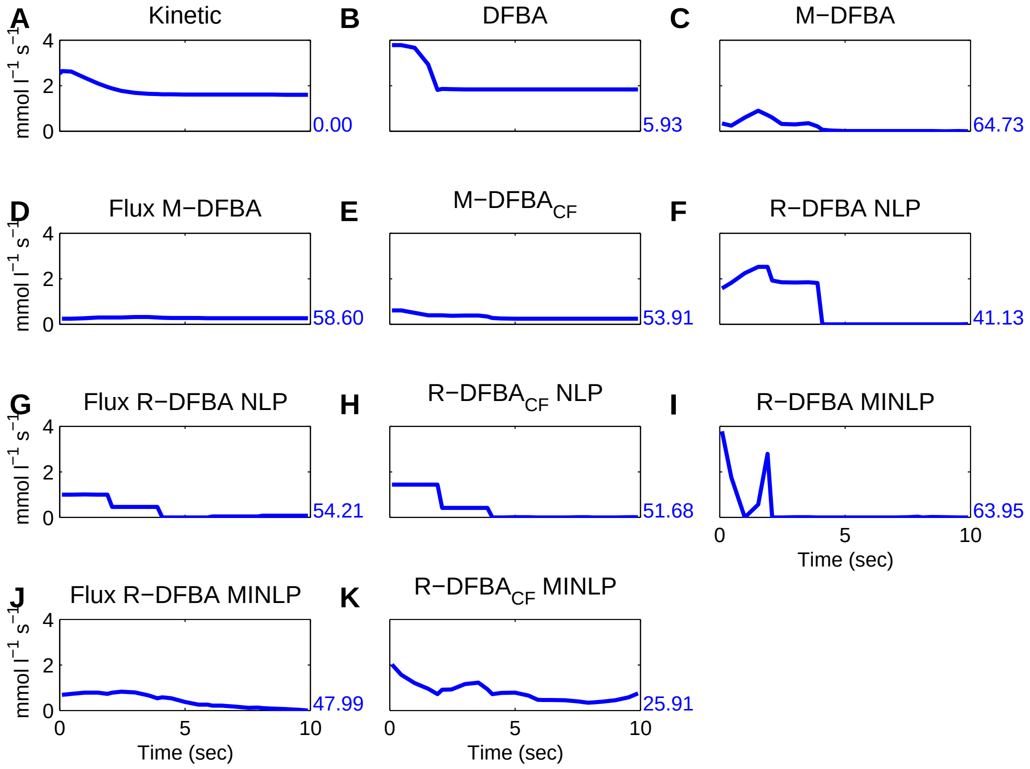

Figure S5: Modeling results of reaction rates of $\mathbf{v}_1$ in the simplified model of the Calvin cycle by the different approaches. The residual sum of squares value of each approach is presented in the bottom right of the corresponding subfigure.

Figure S6

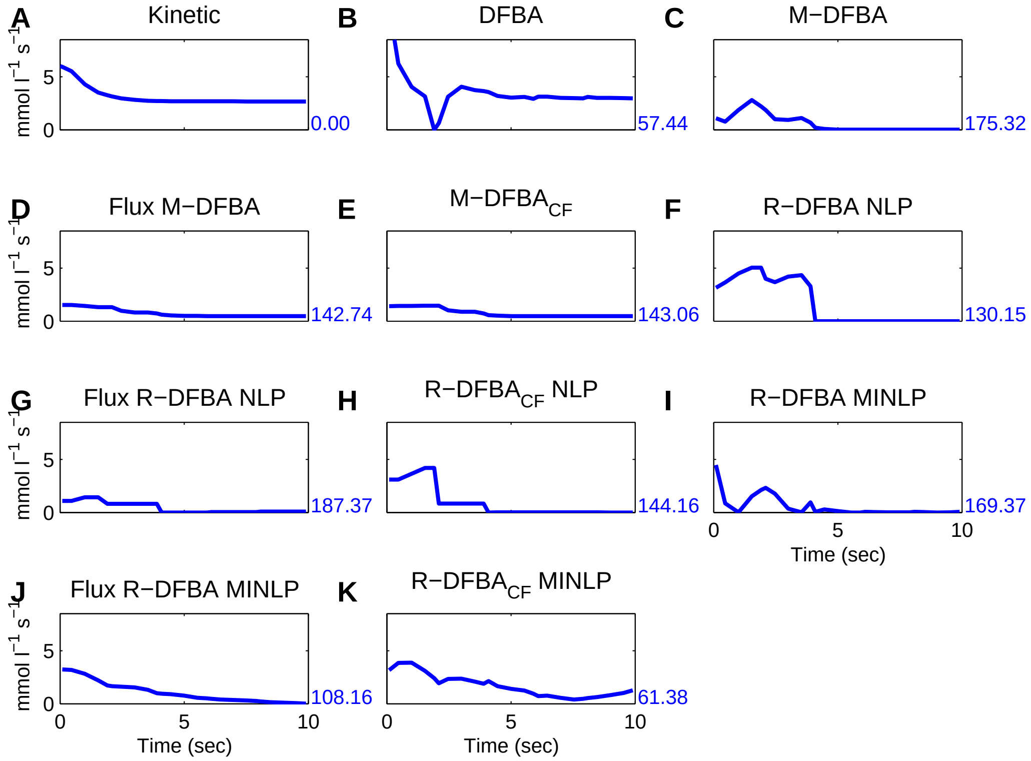

Figure S6: Modeling results of reaction rates of $\mathbf{v}_2$ in the simplified model of the Calvin cycle by the different approaches. The residual sum of squares value of each approach is presented in the bottom right of the corresponding subfigure.

Figure S7

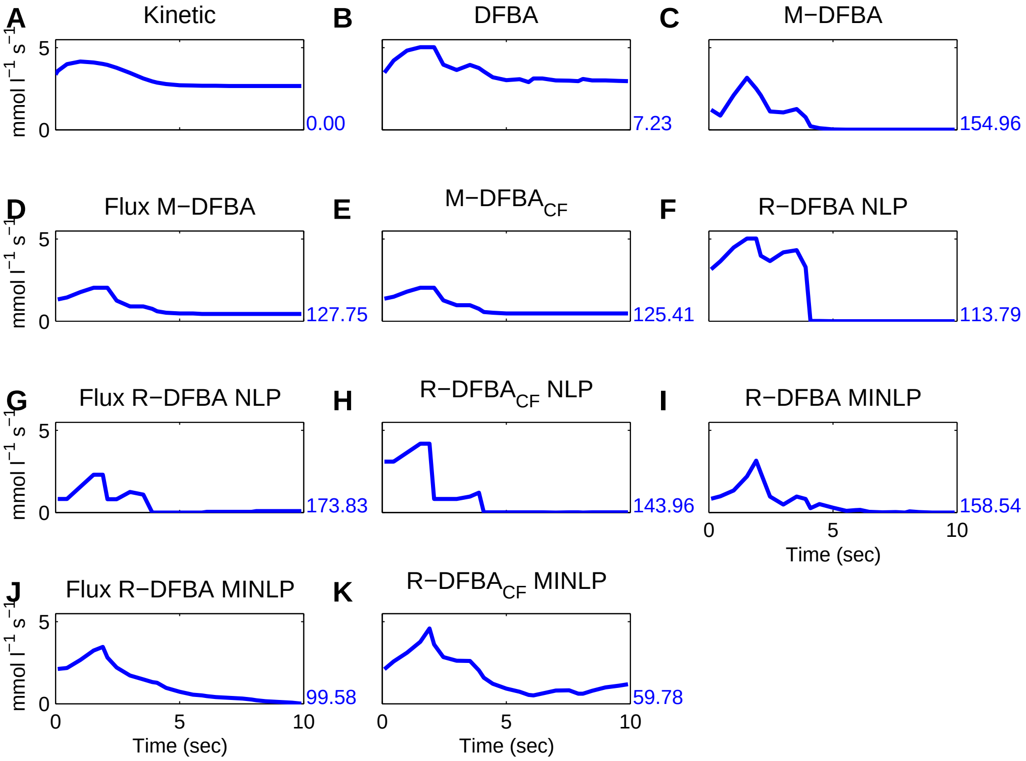

Figure S7: Modeling results of reaction rates of $\mathbf{v}_3$ in the simplified model of the Calvin cycle by the different approaches. The residual sum of squares value of each approach is presented in the bottom right of the corresponding subfigure.

Figure S8

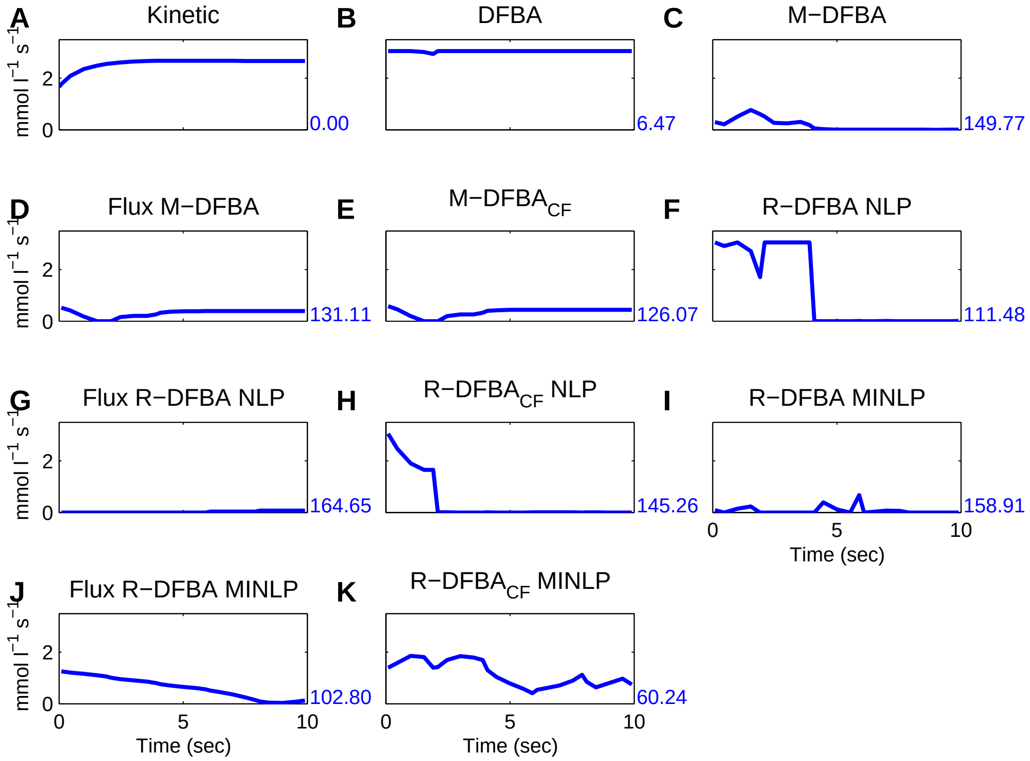

Figure S8: Modeling results of reaction rates of $\mathbf{v}_4$ in the simplified model of the Calvin cycle by the different approaches. The residual sum of squares value of each approach is presented in the bottom right of the corresponding subfigure.

Figure S9

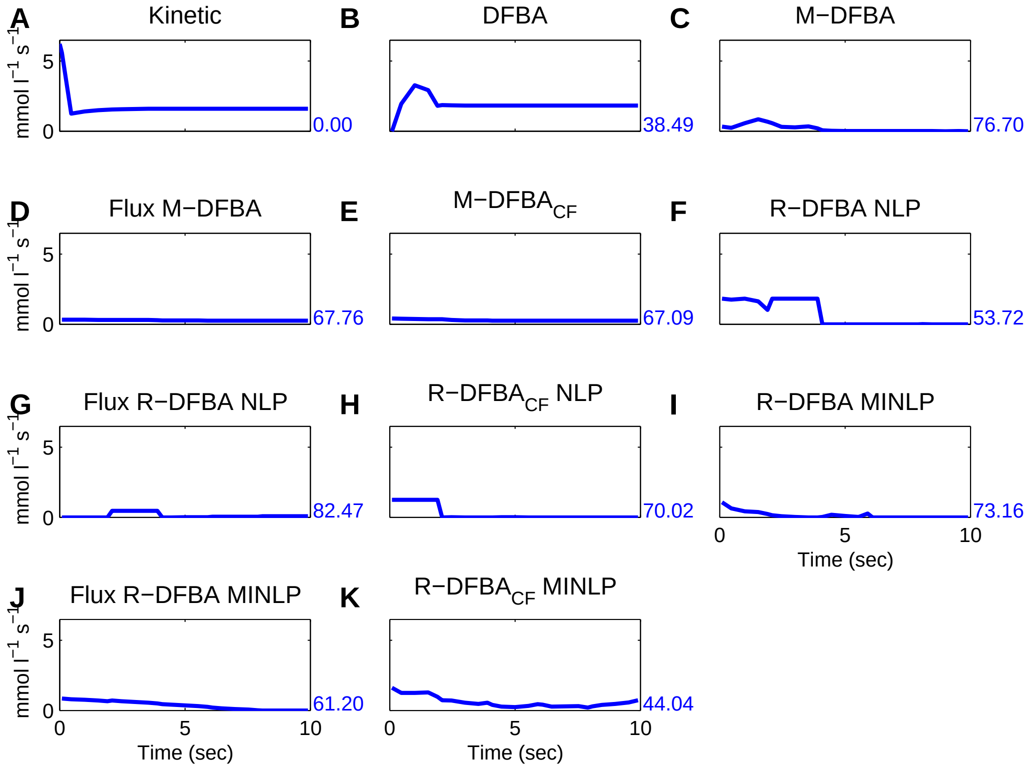

Figure S9: Modeling results of reaction rates of $\mathbf{v}_5$ in the simplified model of the Calvin cycle by the different approaches. The residual sum of squares value of each approach is presented in the bottom right of the corresponding subfigure.

Figure S10

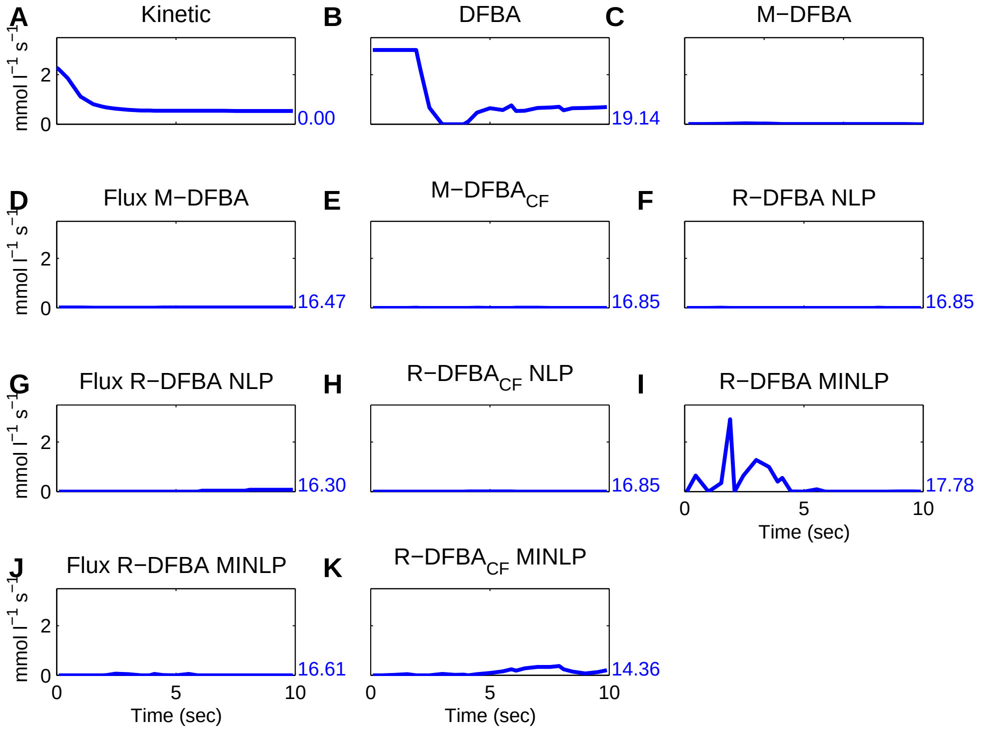

Figure S10: Modeling results of reaction rates of $\mathbf{v}_6$ in the simplified model of the Calvin cycle by the different approaches. The residual sum of squares value of each approach is presented in the bottom right of the corresponding subfigure.

Figure S11

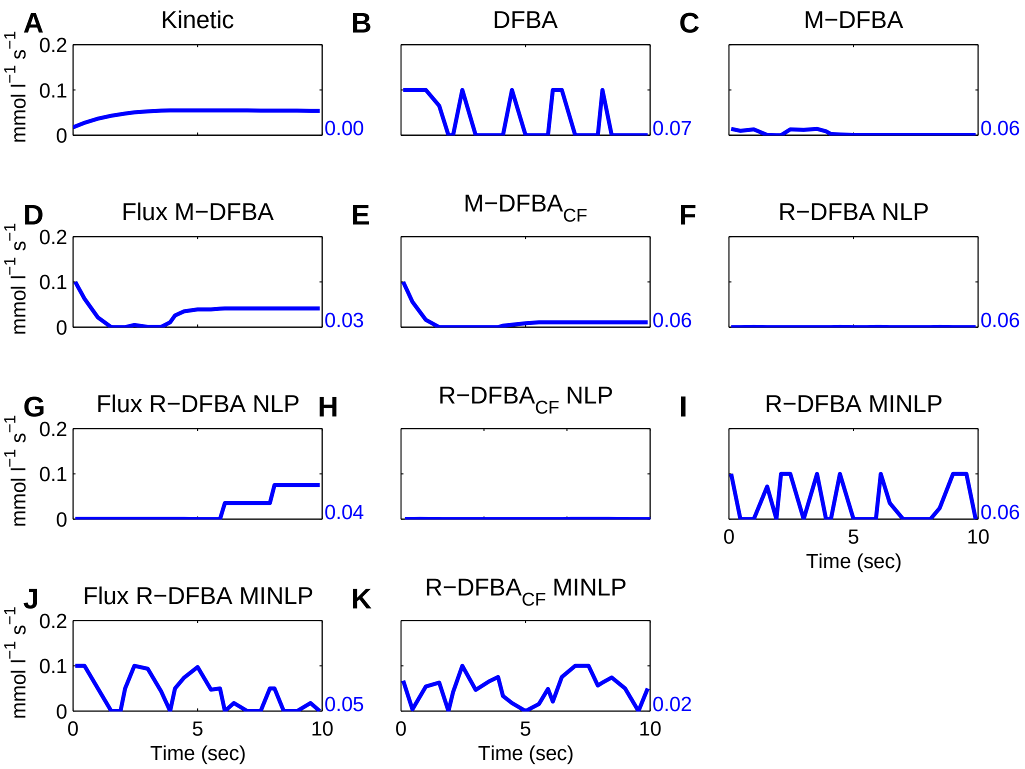

Figure S11: Modeling results of reaction rates of $\mathbf{v}_7$ in the simplified model of the Calvin cycle by the different approaches. The residual sum of squares value of each approach is presented in the bottom right of the corresponding subfigure.
