# Supplementary material for: Dynamic regulatory on/off minimization for biological systems under internal temporal perturbations
Source: BMC Syst Biol. 2012 Mar 12;6:16. doi: 10.1186/1752-0509-6-16 (PMC3361480; doi:10.1186/1752-0509-6-16)
Supplement: Additional file 3 — Additional results Photosynthesis Model. Results from the RSS analysis of the proposed methods for a lumped model of C3-plant carbohydrate metabolism are presented. The model includes 6 metabolites and 7 reactions, as depicted in Figure S12. In addition, the ODEs for the kinetic model used in establishing the RSS results are presented. [file 1752-0509-6-16-S3.PDF]

Model of Central Carbohydrate Metabolism in C_3 plants

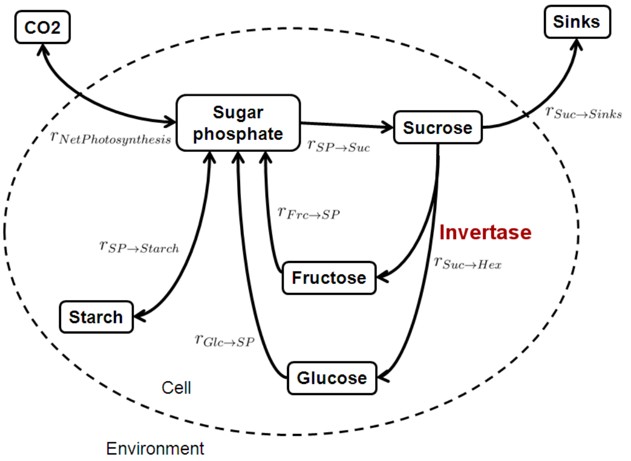

Figure S12: Simplified model of the primary carbohydrate metabolism of plant leaf cells. The invertase reaction ($r_{Suc \to Hex}$) which is shown in red is defected in the *inv4* mutant. Sinks denote a combination of sugar transport to heterotrophic organs and export of assimilated carbon into metabolic pathways.

The model is based on the following system of ordinary differential equations describing time-dependent alterations in carbohydrate pools:

$$\begin{aligned}
\frac{dSP}{dt} &= \frac{1}{6} r_{NetPhotosynthesis} - r_{SP \to Starch} - r_{SP \to Suc} + r_{Fruc \to SP} + r_{Gluc \to SP}, \\
\frac{dStarch}{dt} &= r_{SP \to Starch}, \\
\frac{dSuc}{dt} &= \frac{1}{2} r_{SP \to Suc} - \frac{1}{2} r_{Suc \to Sinks} - r_{Suc \to Hex}, \\
\frac{dGluc}{dt} &= r_{Suc \to Hex} - r_{Gluc \to SP}, \\
\frac{dFruc}{dt} &= r_{Suc \to Hex} - r_{Fruc \to SP}, \\
\frac{dSinks}{dt} &= r_{Suc \to Sinks},
\end{aligned}$$

which include sugar phosphates (SP), sucrose (suc), fructose (fruc), glucose (gluc), starch, hexoses (hex) and sinks. The rate of combined sucrose export

$r_{Suc\rightarrow Sinks}$ depends on the the rate of net photosynthesis $r_{NetPhotosynthesis}$ and the rate of changes in the carbohydrate pools:

$$r_{Suc\rightarrow Sinks} = \frac{1}{6} r_{NetPhotosynthesis} - r_{SP\rightarrow Starch} - r_{sumCH},$$

where r_{sumCH} represents the rate of changes in soluble sugars.

For the reactions $r_{NetPhotosynthesis}$, $r_{SP\rightarrow Starch}$ and r_{sumCH}, v_{min} and v_{max} are chosen from average measured values with an added tolerance of +/- 0.4 $\mu mol\ g^{-1}h^{-1}FW$. The lower and upper boundaries (v_{min} and v_{max}) of the reactions $r_{SP\rightarrow Suc}$, $r_{Suc\rightarrow Hex}$, $r_{Fruc\rightarrow SP}$ and $r_{Gluc\rightarrow SP}$ are set to be $v_{min} = 0$ and $v_{max} = 20$. For the concentrations the upper bounds are set different for the metabolites, c_{max} for glucose, fructose, sucrose and sugar-phosphate are set to 10, whereas the maximal starch and sinks concentrations are set to 50 and 400, respectively. The solutions to MINLP formulation of R-DFBA (and its extensions) are obtained with $\gamma_x, \gamma_v = 0.4$, and $\varepsilon_x, \varepsilon_v = 0.01$, representing the relative and absolute ranges of tolerance, respectively.

Table S1 - Residual sum of squares values for each metabolite in the wild type

The discrepancy between the kinetic modeling results obtained for the wild type plant and the results obtained from the DFBA-based approaches is quantified based on the residual sum of squares.

Method	Metabolite					
	Glucose	Sucrose	Fructose	SP	Starch	Sinks
DFBA	52.76	62.91	16.82	20.53	471.92	2406.58
M-DFBA	47.38	93.34	26.83	6.84	1786.81	6594.19
Flux-based M-DFBA	40.27	50.25	2.85	830.00	69.97	453.60
M-DFBA$_{CF}$	47.78	11.75	19.34	343.81	1748.33	6399.56
R-DFBA NLP	9.12	62.31	0.58	12.69	1806.13	3859.46
Flux-based R-DFBA NLP	39.10	42.94	1.80	460.72	16.63	30.66
R-DFBA$_{CF}$ NLP	9.12	9.64	0.58	2.08	100.87	236.09
R-DFBA MINLP	122.29	33.23	116.22	212.31	576.00	1971.89
Flux-based R-DFBA MINLP	30.16	43.91	182.86	272.58	1073.49	2488.77
R-DFBA$_{CF}$ MINLP	38.35	38.01	340.38	285.24	152.22	730.89

Table S2 - Residual sum of squares values for each reaction in the wild type

The discrepancy between the kinetic modeling results obtained for the wild type plant and the results obtained from the different DFBA-based approaches is quantified based on the residual sum of squares.

**Method**	**Reaction**						
	$r_{SP \to Suc}$	$r_{Suc \to Hex}$	$r_{Fruc \to SP}$	$r_{Gluc \to SP}$	$r_{SP \to Starch}$	$r_{Suc \to Sinks}$	$r_{NetPhotosynthesis}$
DFBA	1111.63	365.24	288.80	563.22	8.91	21.78	8.50
M-DFBA	330.20	92.06	91.34	92.45	9.02	33.96	8.37
Flux-based M-DFBA	291.58	31.86	35.33	32.63	6.68	17.05	7.11
M-DFBA$_{CF}$	208.04	54.77	54.53	37.78	8.59	32.70	7.67
R-DFBA NLP	185.23	47.36	46.73	52.04	9.45	22.31	8.12
Flux-based R-DFBA NLP	1850.81	407.02	415.11	408.16	4.40	9.85	7.46
R-DFBA$_{CF}$ NLP	1425.09	348.59	348.70	356.45	4.06	8.68	7.41
R-DFBA MINLP	4556.17	1167.30	1311.55	1352.15	7.63	22.27	8.05
Flux-based R-DFBA MINLP	8021.63	2316.50	2388.27	2438.52	7.90	19.79	8.34
R-DFBA$_{CF}$ MINLP	6244.61	1830.88	2045.96	1809.93	7.46	20.38	8.52

Table S3 - Residual sum of squares values for each metabolite in the *inv*4 mutant

The discrepancy between the kinetic modeling results obtained for the *inv*4 mutant plant and the results obtained from the DFBA-based approaches is quantified based on the residual sum of squares.

**Method**	**Metabolite**					
	**Glucose**	**Sucrose**	**Fructose**	**SP**	**Starch**	**Sinks**
DFBA	57.39	148.67	114.60	40.50	454.77	2713.54
M-DFBA	33.47	71.55	27.40	19.02	1613.27	5740.82
Flux-based M-DFBA	37.83	77.54	1.25	1063.45	111.83	536.21
M-DFBA$_{CF}$	40.49	12.58	42.09	207.48	1571.49	5651.53
R-DFBA NLP	3.02	18.93	0.12	2.93	1683.30	2853.80
Flux-based R-DFBA NLP	41.01	76.82	1.23	709.54	37.96	57.56
R-DFBA$_{CF}$ NLP	3.02	4.36	0.12	13.92	169.24	332.81
R-DFBA MINLP	39.15	342.94	35.54	194.91	83.44	2305.35
Flux-based R-DFBA MINLP	334.50	108.82	100.45	430.28	330.78	1549.86
R-DFBA$_{CF}$ MINLP	156.38	51.26	273.97	241.93	163.26	698.09

Table S4 - Residual sum of squares values for each reaction in the *inv*4 mutant

The discrepancy between the kinetic modeling results obtained for the *inv*4 mutant plant and the results obtained from the different DFBA-based approaches is quantified based on the residual sum of squares.

**Method**	**Reaction**						
	$r_{SP\to Suc}$	$r_{Suc\to Hex}$	$r_{Fruc\to SP}$	$r_{Gluc\to SP}$	$r_{SP\to Starch}$	$r_{Suc\to Sinks}$	$r_{NetPhotosynthesis}$
DFBA	1349.04	525.79	547.84	382.97	7.91	25.66	8.32
M-DFBA	312.33	82.25	80.84	82.06	9.21	32.46	8.58
Flux-based M-DFBA	363.60	40.69	43.87	49.56	6.24	15.17	7.44
M-DFBA$_{CF}$	1328.11	361.91	360.65	350.27	9.01	32.33	7.72
R-DFBA NLP	293.76	73.91	73.94	74.37	9.35	19.97	8.31
Flux-based R-DFBA NLP	1460.63	297.00	296.91	294.09	5.65	9.22	7.10
R-DFBA$_{CF}$ NLP	1358.52	342.28	342.12	341.11	4.74	6.96	7.24
R-DFBA MINLP	2335.44	601.63	544.88	632.73	8.20	19.03	8.61
Flux-based R-DFBA MINLP	4180.26	1069.43	1066.33	1081.62	6.32	14.38	8.06
R-DFBA$_{CF}$ MINLP	1391.65	455.13	467.39	592.04	6.23	13.13	8.21
